# Hino-Fe Chelate Suppresses Osteosarcoma Progression through Dual Induction of Ferroptosis and NLRC4-mediated Pyroptosis: Mechanisms and Therapeutic Implications

**DOI:** 10.7150/ijbs.113785

**Published:** 2025-07-28

**Authors:** Yuxing Chen, Yong Tao, Qiu Huang, Jingtao Xu, Zhenxin Wang, Ye Zhang, Guangxu Fu, Fuqiang Tan, Keyi Feng, Yunsheng Ou

**Affiliations:** 1 Department of Orthopaedic Surgery, The First Affiliated Hospital of Chongqing Medical University, Yuzhong, Chongqing, 400016, China.; 2 Chongqing Municipal Health Commission Key Laboratory of Musculoskeletal Regeneration and Translational Medicine, Yuzhong, Chongqing, 400016, China.; 3 Orthopaedic Research Laboratory of Chongqing Medical University, Yuzhong, Chongqing, 400016, China.; 4 Department of Orthopaedics, Fengdu General Hospital, Chongqing, 408200, China.

**Keywords:** osteosarcoma, ferroptosis, pyroptosis, hinokitiol-iron complex, NLRC4 inflammasome

## Abstract

Osteosarcoma remains a challenging malignancy with poor prognosis, particularly in metastatic cases. This study investigates the therapeutic potential and molecular mechanisms of the Hinokitiol-iron complex (Fe(hino)_3_) against osteosarcoma. Fe(hino)3 induced dose-dependent cell death in osteosarcoma cell lines (HOS, 143b, and K7M2) through multiple pathways. At moderate doses, Fe(hino)3 triggered ferroptosis by disrupting mitochondrial function, enhancing ROS generation and lipid peroxidation, downregulating GSS and GPX4, and upregulating HO1 and Ferritin expression. At higher doses, Fe(hino)_3_ activated the NLRC4/Caspase-1/GSDMD pathway, leading to pyroptosis and the release of inflammatory factors. Mechanistically, Fe(hino)3 acted as a dual-mode cell death inducer through iron overload-mediated ferroptosis and NLRC4-dependent pyroptosis while modifying the immunosuppressive tumor microenvironment. In actual clinical application, Fe(hino)3 might be used as an alternative to chemotherapy or other targeted therapies for advanced osteosarcoma at a relatively low dose to improve biosafety and reduce side effects. However, when considering it in combination with immunotherapy for advanced osteosarcoma, a relatively safe high dose is more appropriate due to the pyroptosis-mediated inflammatory response but it still needs to consider the biosafety of combination therapy. These findings provide new insights into the development of Fe(hino)3 dose-dependent therapeutic strategies for advanced osteosarcoma treatment.

## Introduction

Osteosarcoma represents the most prevalent primary malignant bone tumor, predominantly affecting pediatric and young adult populations. Despite advancements in multimodal treatment strategies, encompassing surgical intervention, chemotherapy, and radiation therapy 1, the prognosis for individuals with metastatic or recurrent osteosarcoma remains dismal, with a 5-year survival rate of merely 20-30% 2. This highlights the pressing necessity for innovative therapeutic approaches to enhance patient outcomes.

Hinokitiol, a natural tropolone derivative found in the wood of trees belonging to the Cupressaceae family, exhibits diverse biological activities, including antimicrobial, anti-inflammatory 3, and antitumor effects 4. Recent research has investigated the potential of hinokitiol-iron complexes (Fe(hino)3) as innovative therapeutic agents, capitalizing on the distinctive properties of both hinokitiol and iron in modulating cellular processes 5. Compared with traditional anti-tumor drugs, Fe(hino)_3_ has shown its unique advantages. Conventional chemotherapy drugs kill tumor cells mainly by DNA damage. Tumor cells usually rely on high iron levels to maintain rapid proliferation, increasing iron uptake by up-regulating transferrin receptor (TfR1) 6. Fe(hino)_3_ may inhibit tumor growth by competitively binding these transferrins. In addition, Fe(hino)_3_ can directly cause intracellular iron overload, increase reactive oxygen species (ROS) and lipid peroxidation levels, and trigger ferroptosis. Hinokitiol, as an iron carrier, could chelate with irons and transport irons into cells without going through the traditional transferrin 7, thereby inducing ferroptosis in tumor cells 1000 times more efficiently than iron alone 5. Besides ferroptosis, it has also been reported to function through multiple targets and modes (such as down-regulating anti-apoptotic protein BCL-2, up-regulating pro-apoptotic protein BAX 12^8^, inhibiting epidermal growth factor EpCAM expression 9, and regulating the HO-1/Nrf2 antioxidant pathway 10. Moreover, it is effective against chemotherapy-resistant ovarian cancer cells (A2780cis) with a lower IC50 value: 0.92uM. Due to the higher dependence of tumor cells on iron, Fe(hino)_3_ can selectively target tumors with lower toxicity to normal cells such as HEK293^7^. In triple-negative breast cancer animal models, no significant toxicity was observed 5. Iron metabolism plays a crucial role in the function of immune cells, and Fe(hino)3 may affect anti-tumor immunity by altering the iron distribution between tumor cells and immune cells 11, 12. However, the role of Fe(hino)_3_ in osteosarcoma was rarely reported.

Recent investigations suggest that ferroptosis and pyroptosis are highly promising forms of cell death. 13, 14. Ferroptosis, an iron-dependent form of regulated cell death characterized by the accumulation of lipid peroxides, has emerged as a potential vulnerability in various cancer types, including osteosarcoma 15. Key regulators of ferroptosis include glutathione peroxidase 4 (GPX4), glutathione synthesis (GSS), heme oxygenase-1 (HO-1), and ferritin, which collectively modulate cellular iron homeostasis and lipid peroxidation 16, 17. Pyroptosis, a highly inflammatory form of programmed cell death, has garnered significant attention in cancer research due to its potential to enhance anti-tumor immunity 18. This process is mediated by the activation of inflammatory caspases, particularly caspase-1, which facilitates the cleavage of gasdermin D (GSDMD) and the subsequent release of pro-inflammatory cytokines such as IL-1β, IL-18, IL-6, and TNF-α 19. NOD-like receptor Family CARD Domain Containing 4 (NLRC4), an essential member of the NOD-like receptor (NLR) family, possesses a caspase activation and recruitment domain (CARD) 20. Upon activation, NLRC4 directly cleaves caspase-1, which in turn processes the precursors pro-IL-1β and pro-IL-18 and cleaves GSDMD, thereby inducing pyroptosis and inflammatory responses 21. In recent years, NLRC4 has become a focal point in cancer research 22, 23.

The interaction between ferroptosis, pyroptosis, and the immune system offers a promising avenue for cancer therapy 14. Inducing these cell death pathways may not only directly eradicate cancer cells but also stimulate an anti-tumor immune response 24, 25, potentially overcoming the immunosuppressive tumor microenvironment characteristic of osteosarcoma. Some literature has reported that Fe(hino)_3_ can mediate ferroptosis in tumor cells, but there are almost no reports related to pyroptosis 5, 7.

In the present study, we found that in osteosarcoma, relatively low doses of Fe(hino)3 induced ferroptosis, whereas relatively high doses induced the onset of pyroptosis. This dose-dependent modulation is instructive for its clinical use: when used as a single agent, relatively low doses might reduce the side effects of the drug and thus improve biosafety. Then, when used in combination (especially with immunotherapy), relatively high doses may lead to better efficacy due to further activation of immunity induced by pyroptosis, but still needs to consider the biosafety. Although we had observed significant immune activation at relatively safe doses in animals, how to further improve safety for future clinical applications deserves further investigation. By elucidating these mechanisms of Fe(hino)_3_, we aim to provide insights into novel therapeutic strategies for osteosarcoma and potentially other aggressive cancers.

## Materials and Methods

### Reagents and instruments

We obtained Lactate Dehydrogenase (LDH) Activity Assay Kit, Prussian blue, JC1, and 2'7'-dichlorofluorescin diacetate (DCFH-DA) from Solarbio Science & Technology. Bicinchoninic Acid Assay (BCA) and trypsin were sourced from Beyotime Biotech. We purchased Cell Counting Kit-8 (CCK-8) from Dojindo Molecular Technologies. Annexin V-propidium iodide (PI) double-staining test kit was from KeyGen Biotech. Hinokitiol and Deferoxamine (DFO) were sourced from Sigma. Ferric chloride, N-Acetyl-L-cysteine (NAC), Ferrostatin-1 (Fer-1), Belnacasan (VX765), and z-VAD-FMK were obtained from Aladdin.

### Antibodies

Primary antibodies were: glyceraldehyde-3-phosphate dehydrogenase (GAPDH) (1:3000), cleaved caspase-3 (1:1000), cleaved caspase-1(1:1000), cleaved poly ADP-ribose polymerase (PARP) (1:1000), B-cell lymphoma-2 (Bcl-2) (1:1000), BCL2 Associated X (Bax) (1:1,000), HO-1 (1:1000), NADH: Ubiquinone Oxidoreductase Core Subunit S2 (NDUFS2) (1:1000), Succinate Dehydrogenase Complex Flavoprotein Subunit A (SDHA) (1:1000), GSS (1:500), Ferritin (1:1000), Secondary antibodies were: Horseradish Peroxidase (HRP) monoclonal antibody anti-IgG of mouse and HRP monoclonal antibody anti-IgG of rabbit; all from ZEN-BIOSCIENCE, Chengdu, China), NRLC4((1:1000, Bioworld Technology, Inc.), GSDMD-N(1:1000), CD11C(1:100), CD86(1:100), CD4(1:100) and CD8(1:100; all from HUABIO).

### Cell lines and culture

The K7M2, HOS, and 143b cell lines were obtained from Wuhan PriCell Biotechnology Co., Ltd. (China) and subsequently cultivated in Dulbecco's Modified Eagle's Medium (DMEM). This was further enhanced by incorporating 10% fetal bovine serum (FBS) obtained from HyClone, a manufacturer based in Wuhan, China. In addition to the 100 µg/mL penicillin and streptomycin provided by Beyotime Biotech, the solution also contains these antibiotics at the specified concentrations. To ensure optimal cellular function, the culture environment was carefully controlled by keeping the cells at a temperature of 37°C within a controlled atmosphere that also included a precise 5% concentration of carbon dioxide.

### Synthesis of Fe(hino)_3_

The synthesis of Fe(hino)_3_ was carried out according to a previously established method 5. Specifically, iron (III) chloride (560.2 mg, 2.08 mmol) and hinokitiol (1.026 g, 6.24 mmol) were dissolved in 10 mL of ethanol and the resulting solution was stirred vigorously at room temperature for 2 hours, forming a cherry red-purple suspension. Then the supernatant was aspirated off and the precipitate was retained. Fe(hino)_3_ in solid form was re-solubilized by anhydrous ethanol and was allowed to stand at room temperature for 10 minutes. The supernatant was aspirated off when Fe(hino)_3_ was sufficiently precipitated. This process was repeated 3 times to remove residual iron (III) chloride and hinokitiol. Finally, the residual product was isolated by filtration, dried in a fume hood at 60℃ for 15 minutes, and yielding the purple solid Fe(hino)3. The compound was subsequently characterized using spectrometric techniques, scanning electron microscope (SEM), X-ray, etc.

### LPO measurements

Following a 24-hour incubation period, osteosarcoma cells were carefully introduced into 6-well plates at a concentration of 1 × 10^5 cells per well, with the aim of enabling them to form stable cell monolayers. After the respective treatments, a 1:500 dilution of a C11-Bodipy working solution was introduced and allowed to incubate at 37 °C for 30 minutes in the dark. The cells were then analyzed under a fluorescence microscope for detailed imaging, or alternatively, they underwent flow cytometry to assess their markers.

### LDH release assays

After applying a series of therapeutic interventions, the supernatant from the cultured cells was carefully harvested and subjected to centrifugation at 3000 revolutions per minute for a duration of ten minutes. Then, 120 μl of the supernatant was carefully pipetted into each well of a 96-well microplate. To ensure reliable data collection, three identical wells were constructed for each experimental group, and these samples were then subjected to analysis using the LDH release assay kit provided by Beyotime. The absorbance values were determined at a wavelength of 490 nm using a microplate spectrophotometer.

### siRNA transduction

During the logarithmic growth phase of osteosarcoma cell proliferation, we opted to perform transfection at a confluence level of approximately 50% to ensure optimal cellular viability and enhance transfection efficiency. At a 100 nM concentration, three distinct siRNA molecules (siRNA-1, siRNA-2, and siRNA-3) were utilized to specifically target the GSDMD gene, with their respective sequences as follows: siRNA-1: 5'-gtgtcaacctgtctatcaagg-3', siRNA-2: 5'-cgctgagtgtggaccctaaca-3', and siRNA-3: 5'-ggcagactctgctccatgaga-3'. The sequences of siRNA-1, siRNA-2, and siRNA-3 for NLRC4 were selected as follows: siRNA-1 was 5'-gcaaatcacagatgacctatt-3', siRNA-2 was 5'-gggatttcagcaagttgaata-3', and siRNA-3 was 5'-ggatgaacgtgctagaacagc-3'. These siRNA sequences were then synthesized using Lipofectamine-2000 to create a transfection complex in a serum-free medium, which was subsequently diluted to form the final transfection reagent. The complex was systematically incorporated into the cellular environment, and the culture plate was carefully agitated to promote even distribution of the substance throughout the medium. After a 6-hour incubation period, the supernatant was carefully collected, and the cells were then reconstituted using a fresh complete medium to ensure optimal growth conditions. Following the initial cultivation phase, the cells underwent a further 72-hour incubation period to allow for complete adaptation and proliferation, before the initiation of the next series of experimental procedures.

### Intracellular iron ion detection

During the logarithmic growth phase, osteosarcoma cells were introduced into culture wells at a concentration of 2 × 10⁴ cells per well. After a 48-hour incubation and the completion of the prescribed therapeutic interventions, the levels of intracellular iron were assessed through the Prussian Blue Test Kit, following the manufacturer's detailed procedures.

### CCK-8 assay and treatment

Osteosarcoma cells underwent a trypsinization process before being transferred into 96-well plates, with each well containing a density of 5,000 cells. After a 48-hour incubation, the culture medium was replaced with a fresh solution that contained different levels of Fe(hino)3 (0, 0.5, 1.0, 2.0, 4.0, 6.0 µM for the 143B, HOS, and K7M2 cell lines) or Hinokitiol (0, 10, 20, 30, 40, 50, 60 µM for the same cell lines). Following a 24-hour incubation, a volume of 10 μL from the CCK-8 working solution was added to each well. Subsequently, the sample underwent a 2-hour incubation process at a controlled temperature of 37 °C under strictly light-avoiding conditions. The absorbance value at 450 nm was quantified through a microplate reader system. The assessment of cell survival was conducted through the application of this mathematical equation: Cell viability (%) = [(OD of the experimental group - OD of the blank control) / (OD of the control - OD of the blank control)] × 100%.

### Western blot analysis

After undergoing a series of experimental interventions, osteosarcoma cells were lysed through the application of Radio Immunoprecipitation Assay (RIPA) buffer containing 1% Phenylmethylsulfonyl fluoride (PMSF) and 1% phosphatase inhibitors. Following this, the sample was then placed at ice for 30 minutes. After the centrifugation process was completed, the upper liquid layer, which is known as the supernatant, was carefully gathered for subsequent analysis. To determine the protein content within this sample, the BCA (bicinchoninic acid) assay was employed as a reliable method for quantification. To analyze the protein composition of the samples, a quantity of 40 μg was taken from each test case and then subjected to Sodium Dodecyl Sulfate-Polyacrylamide Gel Electrophoresis (SDS-PAGE) under a consistent voltage range of 80-100 volts. Subsequently, proteins were immobilized on polyvinylidene fluoride (PVDF) membranes by applying a steady electric current of 250 mA. The transfer process is optimized by aligning the duration of the transfer with the molecular weight characteristics of the target proteins. To ensure effective membrane permeabilization, we employed a 5% skim milk solution in Tris Buffered Saline Tween (TBST) for a duration of two hours at ambient temperature. Prior to the incubation, primary antibodies were incubated at 4 °C for a period of overnight, with gentle mixing, and then subjected to thorough washing using TBST buffer. Following this, the secondary antibodies, which were prepared at a concentration of 1:5000, underwent a 2-hour incubation at room temperature under continuous stirring. The protein detection process was executed via chemiluminescence imaging, and subsequent photographic records were meticulously recorded. The analysis of grayscale intensity values from the bands was conducted using Image J software to assess relative protein expression levels, with GAPDH serving as an internal reference for normalization.

### Measurement of ROS

Cells were cultivated in both 6-well and 24-well culture plates, with the 6-well plates containing 1×10⁵ cells per well and the 24-well plates accommodating 5×10⁴ cells per well. Following the completion of the prescribed therapeutic protocols, the experimental cells were subjected to a controlled incubation period that extended beyond the initial 4 hours. Following this, the compound DCFH-DA was administered at a concentration of 10 µM and allowed to remain in the incubation chamber at a controlled temperature of 37°C for a duration of 20 minutes. Ultimately, the cells within the 24-well plates underwent three rounds of washing and were then analyzed via fluorescence microscopy (FM). During the process of preparing the cells for analysis, they were first subjected to enzymatic digestion using trypsin, then carefully separated and gathered into individual wells within a 6-well plate, before undergoing flow cytometric examination.

### MMP assay

To assess the mitochondrial membrane potential (Δψm), a 5,5′,6,6′-Tetrachloro-1,1′,3,3′-tetraethyl-imidacarbocyanine iodide (JC-1) assay kit was employed, which is based on the fluorescent dye JC-1.To study the behavior of osteosarcoma cells, we seeded them into 6-well plates at a concentration of 1 × 10⁵ cells per well and maintained them in incubation for a night to facilitate proper cell adhesion. Following the treatment phase, a JC-1 staining solution was introduced into each well and allowed to remain for 20 minutes in an environment free from direct light exposure. Following the initial incubation step, the cells were carefully rinsed multiple times to remove residual substances, and after this cleaning process, they were either directly visualized under a fluorescence microscope or processed for further examination using a flow cytometer.

### RNA sequencing

After undergoing the steps of digestion, washing, and centrifugation as part of a comprehensive treatment regimen for osteosarcoma cells, The TRIzol reagent was prepared at a concentration of 5 × 10^6 cells per milliliter to ensure optimal extraction efficiency. The final mixture was then carefully moved into a sealed, RNase-free cryogenic storage container. Following this, the samples underwent a controlled cooling process in liquid nitrogen for a duration of 30 minutes to ensure their rapid freezing. Following the freezing process, the tubes were individually enclosed in self-sealing plastic containers to prevent any leakage and then kept at a controlled temperature of -80 °C for extended storage. Following the completion of the experimental procedures, the collected samples were immediately sent to Majorbio Co. (China) for transcriptome sequencing, during which we performed comprehensive analyses to identify differentially expressed genes and elucidate their biological significance by examining enriched pathways.

### Immunohistochemical staining

The tumor tissue samples underwent a two-hour baking treatment, after which they were deparaffinized with a sequence of xylene and ethanol. The antigen retrieval process was executed in an Ethylenediaminetetraacetic acid (EDTA) buffer, with the sample subjected to microwave heating at high intensity for a duration of five minutes. The procedure began with a low-power setting, which was then gradually increased to a medium level, and this adjusted intensity was maintained for a duration of 15 minutes. After the cooling process was completed, the sections underwent treatment with a 0.3% hydrogen peroxide solution to suppress the activity of peroxidase enzymes, followed by a blocking step that lasted for twelve minutes. A primary antibody solution, prepared by diluting the original stock at a concentration of 1:500, was introduced into the experimental setup and allowed to remain at a temperature of 4°C for an extended period of time. Following a complete cleansing process, the researchers then added an additional detection antibody and allowed it to remain at 37 °C for a duration of two hours. Following diaminobenzidine (DAB) development, the specimens underwent a series of critical staining and processing steps, beginning with hematoxylin-based counterstaining to enhance contrast. Subsequently, they were carefully dehydrated using an alcohol gradient, followed by clearing agents to preserve their structural integrity, and finally prepared for mounting in a suitable medium to enable microscopic analysis.

### Hematoxylin-eosin (H&E) staining

The tissue samples were first immersed in a 4% paraformaldehyde solution for a period of 24 hours to ensure proper preservation, followed by their embedding within paraffin wax to facilitate the creation of histological sections, which were then cut down to a uniform thickness of 4 micrometers. After completing the deparaffinization process and rehydration using a stepwise alcohol protocol, histological analysis was performed using hematoxylin and eosin (HE) staining as per standard laboratory procedures. The designated segments underwent a thorough treatment process and were then affixed in a stable, non-reactive resin-based solution to safeguard their integrity for long-term storage. A detailed morphological analysis of the specimen was conducted using a Nikon Eclipse brightfield microscope, which enabled precise observation under controlled lighting conditions.

### Xenograft tumor model

All animal studies were approved by the Institutional Animal Care and Use Committee of Chongqing Medical University (IACUC-CQMU-2024-0472). A total of 1 × 10^6^ HOS cells, suspended in 100 μL of DMEM, were subcutaneously injected into 6-week-old female nude mice, while an equivalent number of K2M2 cells in the same volume were administered to 6-week-old female Balb/c mice. Treatment commenced when tumors reached an approximate volume of 70 mm³. The mice were allocated into two treatment cohorts: one receiving a vehicle and the other receiving 50 μg of Fe(hino)3 per dose directly into the tumors. The same dose (50 ug) was again administered via an intratumoral injection after 5 days. No more injections after that. The injectable dose of Fe(hino)3 in the breast cancer model was 40 ug, and the second dose was given 5 days later 5. Considering that relatively high doses of Fe(hino)3 induced a more pronounced pyroptosis *in vitro* experiments, Therefore, in *in vivo* experiments, the dose of a single intratumoral injection was increased to 50 ug and the second shot was also given after 5 days. To further assess the biosafety of the Fe(hino)3, the administration of three injections (each 50ug and 5 days apart) was evaluated. In addition, to assess the maximum tolerated dose of a single injection, 100 ug, 150 ug, 200 ug, and 250 ug administration doses were explored. Tumor volume was assessed tri-daily using the formula: volume = 1/2 × (short diameter)² × (long diameter). At the conclusion of the study, the mice were euthanized, and tumor measurements were documented.

### Determination of serum iron

80 ul of blood was collected from mice at various time intervals: 0 h, 12 h, 24 h, 48 h, 132 h, 144 h, 168 h, 288 h, and 480 h. The whole blood was allowed to clot for 2 hours, followed by centrifugation at 3000 rpm for 15 minutes; thereafter, the upper serum layer was carefully aspirated. The serum was then preserved in a freezer at -80 ℃. The serum was diluted and quantified to 100ul. For the standard curve, a gradient dilution of iron ions was prepared at concentrations of 50, 25, 12.5, 6.25, and 3.125 umol/L. In the blank wells, 100 ul of assay buffer was added separately, while 100 ul of the sample to be tested was introduced into the respective sample wells. Subsequently, each sample well received an additional 100 ul of assay buffer and was incubated at 37 ℃. Following a 40-minute incubation period, centrifugation was carried out at 10,000 g for 5 minutes. Two hundred ul of the supernatant was then transferred to the enzyme plate, where the absorbance was measured at 593 nm using the enzyme marker. A standard curve was constructed to determine the total iron concentration of the samples. The equation used to estimate the drug blood concentration is Cx = Ch (total iron concentration at the specified time) - C0 (total iron concentration at 0 h).

### Prussian blue staining

Paraffin slices were first immersed in xylene I for 20 minutes, followed by xylene II for an additional 20 minutes. Next, they underwent a treatment with anhydrous ethanol I for 5 minutes, and then with anhydrous ethanol II for another 5 minutes, before being placed in 75% alcohol for 5 minutes. After this, the sections were washed three times with tap water and distilled water. A mixture of Prussian blue dye solution A and Prussian blue dye solution B was prepared in a 1:1 ratio to create the Prussian blue dye solution, into which the slices were stained for 1 hour before being rinsed with distilled water twice. The nuclei were then stained using Prussian Blue Stain Solution C for 3 minutes, followed by a rinse under running water. Subsequently, the sections were processed in anhydrous ethanol I for 5 minutes, anhydrous ethanol II for 5 minutes, and anhydrous ethanol III for 5 minutes, before undergoing a treatment with xylene I for 5 minutes and xylene II for 5 minutes for transparency. Finally, the slices were mounted with neutral gum, and microscopic examination was conducted, with images being captured.

### Visceral iron content assay

36 hours after intratumoral injection of Fe(hino)_3_, the mice were euthanized, and organs such as the heart, liver, spleen, lungs, and kidneys were carefully extracted. The visceral tissues underwent complete digestion with an acidic solution and were then diluted to a final volume of 10 ml. Standard solutions were formulated based on concentration gradients of 0.5 mg/L, 1 mg/L, 3 mg/L, 5 mg/L, and 10 mg/L. The iron content in the standard solutions was analyzed using Inductively Coupled Plasma Mass Spectrometry (ICP-MS), which facilitated the creation of a standard curve. The samples being investigated were analyzed on the ICP-MS device, and the iron concentrations were calculated using the established standard curve.

### Statistical analysis

The data was presented in the form of the mean ± standard deviation (SD), and they were subjected to statistical analysis employing SPSS software, which is a widely used program developed by SPSS Inc. (Chicago, IL, USA). To evaluate the disparities among different groups, we employed either one-way or two-way analysis of variance (ANOVA) as appropriate, and when comparing two groups, we utilized independent-sample t-tests. Paired t-test was used for comparing differences between paired samples. In scientific research, a p-value threshold of 0.05 is typically used as a criterion to determine whether an observed result is statistically significant, meaning that the probability of obtaining such results by chance alone is less than 5%.

## Results

### Fe(hino)_3_ inhibited osteosarcoma cell proliferation and induced cell death

**Figure 1:**
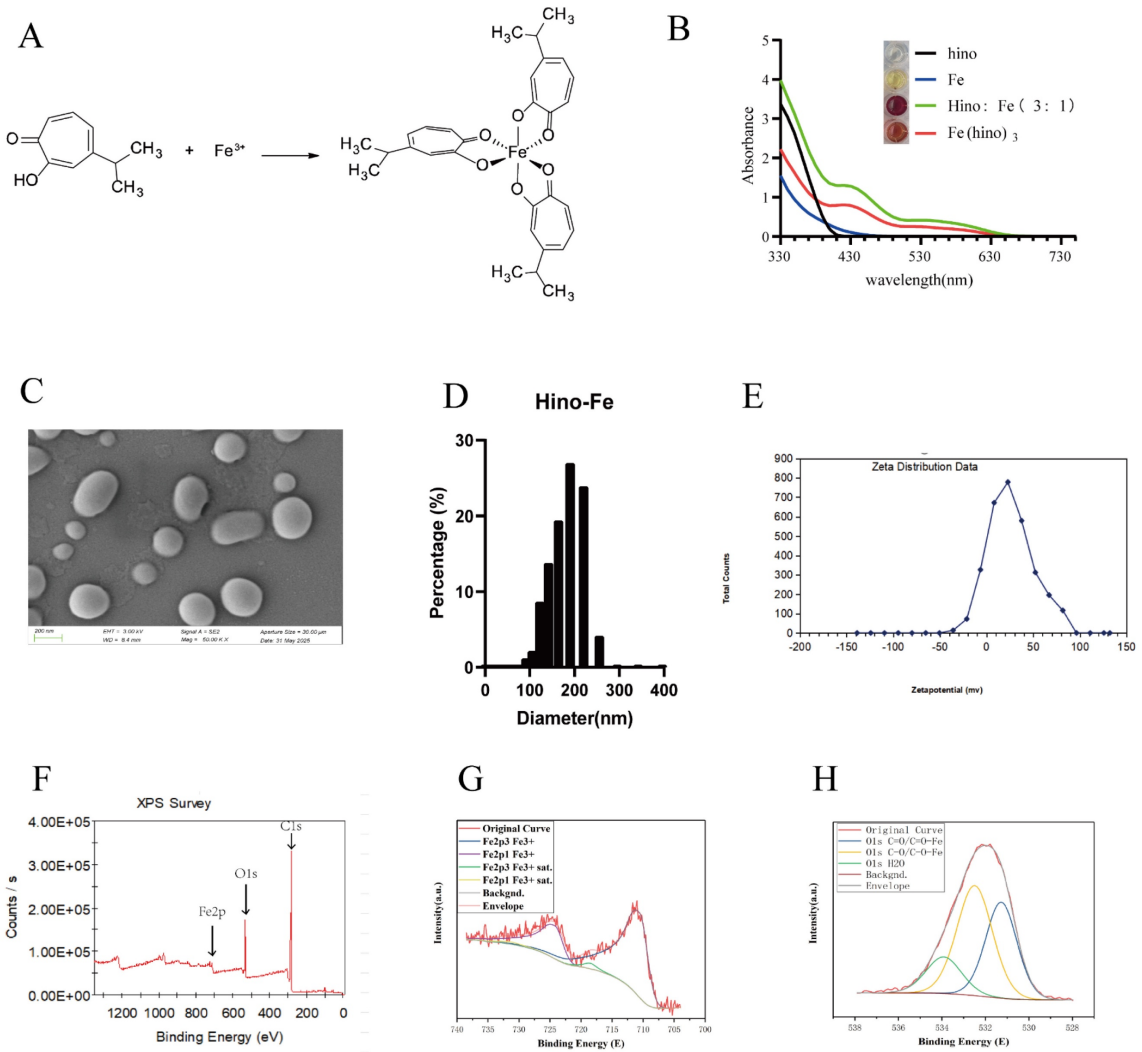
** Material characterization of Fe(hino)3. (A)** Chemical structure of Fe(hino)3 complex. **(B)** UV-vis absorption spectra of hinokitiol, FeCl_3_, and Fe(hino)3. **(C)** Scanning electron microscopy of Fe(hino)3. **(D)** DLS of Fe(hino)3. **(E)** Zeta potential of Fe(hino)3. **(F-H)** XPS results of Fe(hino)3.

The Fe(hino)_3_ complex was successfully synthesized, as depicted in Figure 1A, and its structural integrity was verified through UV absorption spectroscopy, shown in Figure 1B. SEM showed that Fe(hino)_3_ was a spherical particle (Figure 1C). Dynamic Light Scattering (DLS) suggested that Fe(hino)_3_ was mainly distributed around 200 nm, specifically, 70.221% of the particles were distributed in the range of 164.183 nm-220.194 nm. The zeta potential of Fe(hino)_3_ was mainly concentrated around 22 mv (Figure 1E). X-ray photoelectron spectroscopy (XPS) showed that the synthesized Fe(hino)_3_ includes Fe, C and O elements (Figure 1F). The peak fitting of Fe suggested that the Fe elements in Fe(hino)_3_ were all at +3 valence (Figure 1G). The peak fitting of the O element suggested that Fe(hino)_3_ contains C=O-Fe and C-O-Fe bonds in close ratio (Figure 1H).

**Figure 2:**
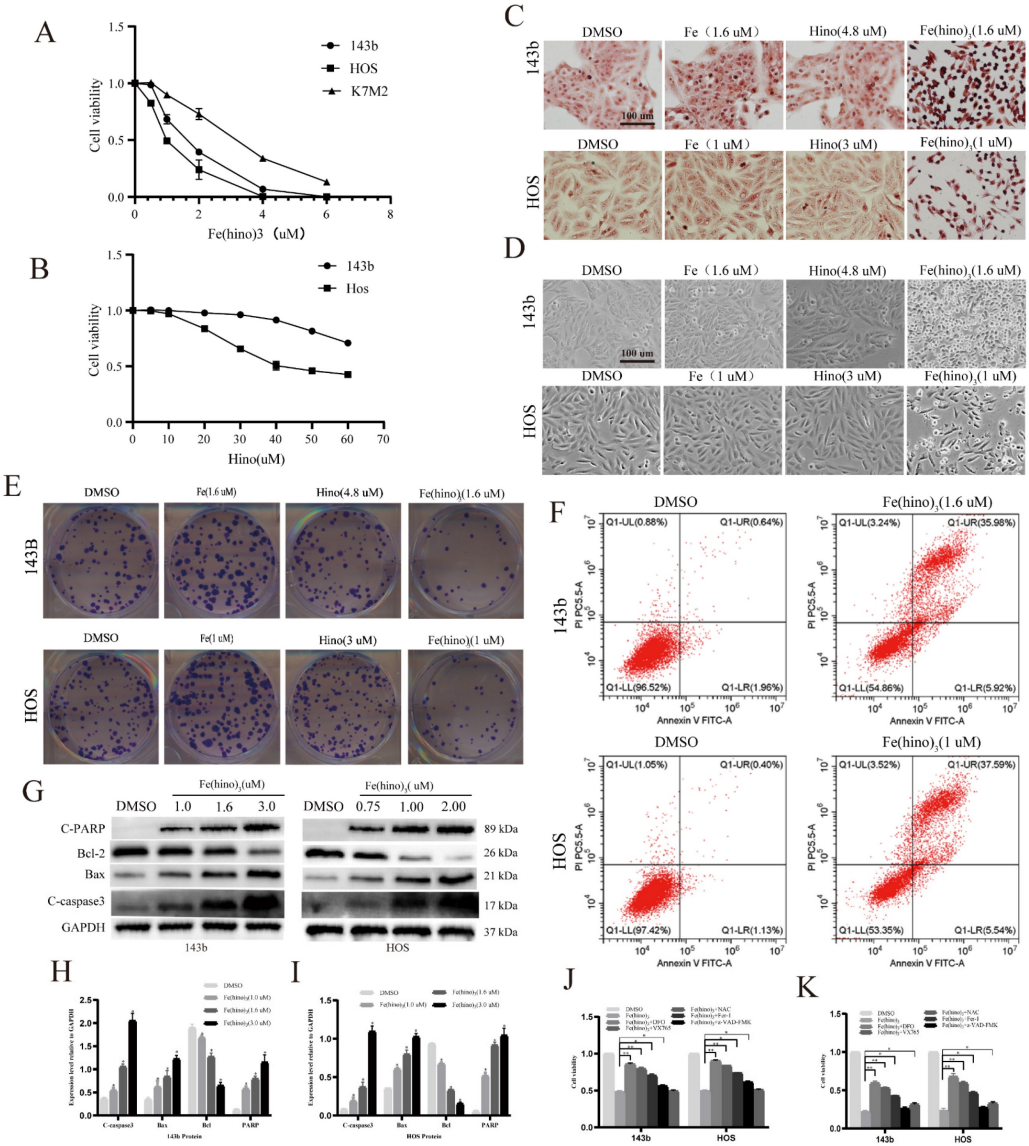
** Fe(hino)3 inhibited osteosarcoma cell proliferation and promoted cell death. (A)** Cell viability of osteosarcoma cells treated with different concentrations of Fe(hino)_3_ (0, 0.5, 1.0, 2.0, 4.0, 6.0 μM) for 24 h, as measured by CCK-8 assay. **(B)** Cell viability of osteosarcoma cells treated with different concentrations of hinokitiol (0, 5, 10, 20, 30, 40, 50, 60 μM) for 24 h. **(C)** Prussian blue staining showing intracellular iron accumulation after treatment with FeCl3 (1.6 μM for 143b, 1 μM for HOS), hinokitiol (4.8 μM for 143b, 3 μM for HOS), or Fe(hino)3 (1.6 μM for 143b, 1 μM for HOS) for 24 h. Scale bar = 100 μm. **(D)** Morphological changes of osteosarcoma cells after treatment with indicated compounds for 24 h. Scale bar = 100 μm. **(E)** Colony formation assay showing the long-term effects of Fe(hino)3 on osteosarcoma cell proliferation. **(F)** Flow cytometry analysis of cell apoptosis using Annexin V/PI staining after Fe(hino)3 treatment for 24 h. **(G-I)** Western blot analysis and quantification of apoptosis-related proteins after Fe(hino)3 treatment (1, 1.6, 3 μM for 143b; 0.75, 1, 2 μM for HOS) for 24 h. **(J, K)** Cell viability after treatment with Fe(hino)_3_ in combination with different cell death inhibitors. Cells were pretreated with DFO (50 μM, 6 h), NAC (5 mM, 1 h), Fer-1 (1 μM), z-VAD-FMK (25 μM, 1 h), or VX765 (30 μM, 1 h) before Fe(hino)_3_ treatment. Data are presented as mean ± SD from three independent experiments. *P < 0.05, **P < 0.01 vs control group.

CCK-8 assays demonstrated that Fe(hino)_3_ inhibited the proliferation of osteosarcoma cell lines (143b, HOS, and K7M2) in a dose-dependent manner (Figure 2A), with significantly lower IC50 values compared to hinokitiol alone (Figure 2B). Prussian blue staining revealed considerable intracellular iron accumulation in osteosarcoma cells following a 24-hour treatment with Fe(hino)3 (Figure 2C). Morphological analysis indicated that cells treated with Fe(hino)3 exhibited pronounced retraction and rounding (Figure 2D). Additionally, colony formation assays corroborated the ability of Fe(hino)3 to suppress the clonogenicity of osteosarcoma cells (Figure 2E). Flow cytometry analysis revealed a significant increase in apoptosis among Fe(hino)3-treated cells (Figure 2F), which was further supported by Western blot analysis showing a dose-dependent upregulation of apoptosis-related proteins, including cleaved-PARP, cleaved-caspase3, and BAX, alongside a downregulation of the anti-apoptotic protein BCL2 (Figure 2G-I).

### Iron and oxidative stress mediated Fe(hino)3-induced cell death

To elucidate the mechanisms underlying Fe(hino)3-induced cytotoxicity, inhibitors targeting key pathways were employed. At moderate concentrations (IC50), the DFO, ROS scavenger NAC, and ferroptosis inhibitor Fer-1 significantly mitigated Fe(hino)3-induced cell death, whereas the caspase-3 inhibitor Z-VAD-FMK provided only partial protection (P < 0.05) (Fig. 2J). Notably, at higher concentrations (IC80), DFO, NAC, and Fer-1 maintained their protective effects, while the partial efficacy of Z-VAD-FMK diminished (P > 0.05). Conversely, the caspase-1 inhibitor VX765 significantly reduced cytotoxicity under high-dose conditions (P < 0.05) (Fig. 2K).

### Fe(hino)_3_ disrupted mitochondrial function and triggered ferroptosis

**Figure 3:**
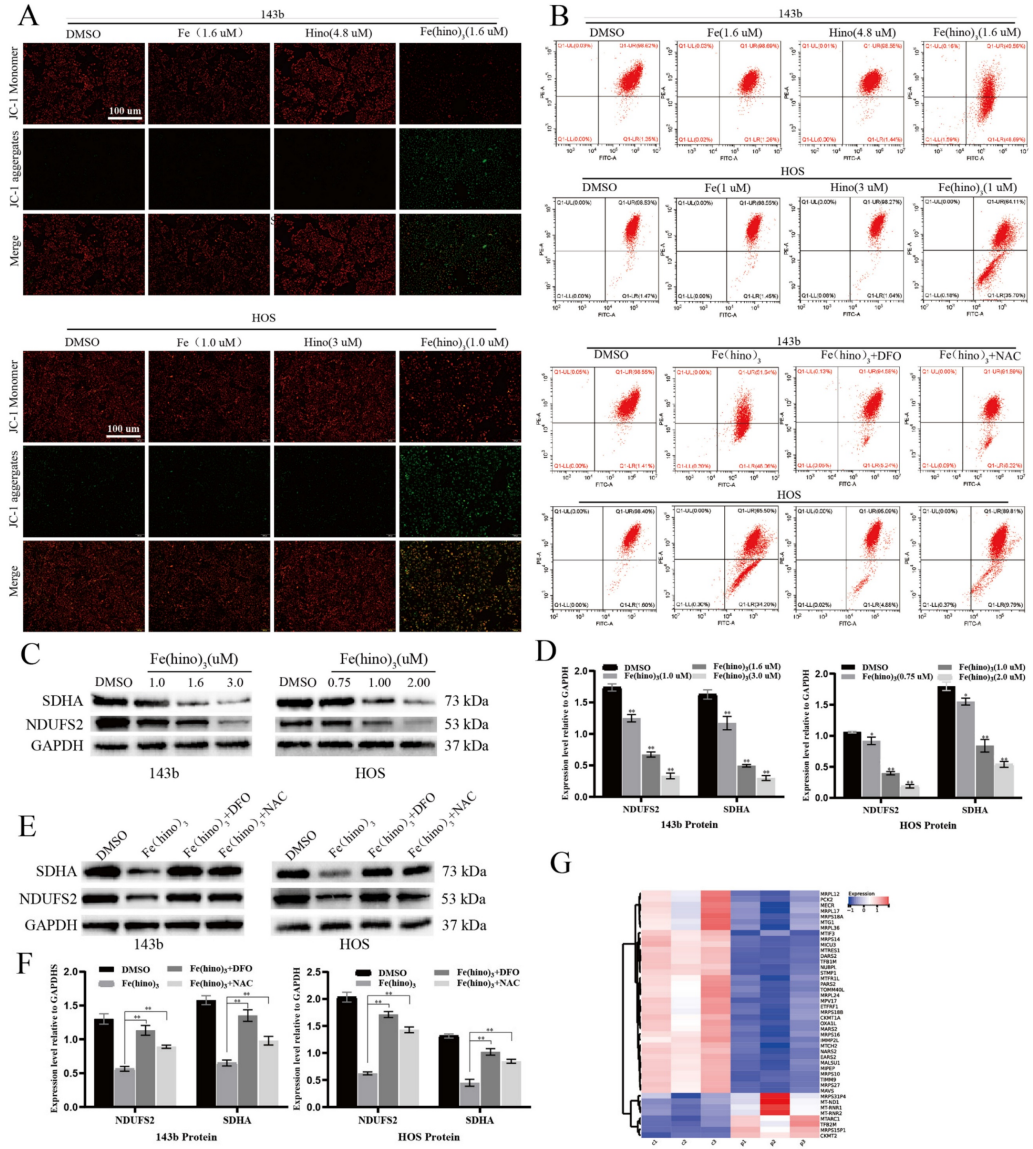
** Fe(hino)_3_ induced mitochondrial dysfunction in osteosarcoma cells. (A)** JC-1 staining showing mitochondrial membrane potential changes after treatment with indicated compounds for 8 h. Scale bar = 100 μm. **(B)** Flow cytometric analysis of JC-1 staining. The effects were reversed by pretreatment with DFO (50 μM, 6 h) or NAC (5 mM, 1 h). **(C-D)** Western blot analysis and quantification of mitochondrial respiratory chain complex proteins after Fe(hino)3 treatment (1, 1.6, 3 μM for 143b; 0.75, 1, 2 μM for HOS) for 24 h. **(E-F)** Western blot analysis showing the protective effects of DFO (50 μM, 6 h) and NAC (5 mM, 1 h) pretreatment on Fe(hino)3-induced changes in mitochondrial proteins. **(G)** RNA-seq analysis showing downregulation of mitochondria-related genes after Fe(hino)3 (1.6 μM) treatment for 24 h in 143b cells. Data are presented as mean ± SD from three independent experiments. P represents an exposure of Fe(hino)3. *P < 0.05, **P < 0.01 vs control group.

**Figure 4:**
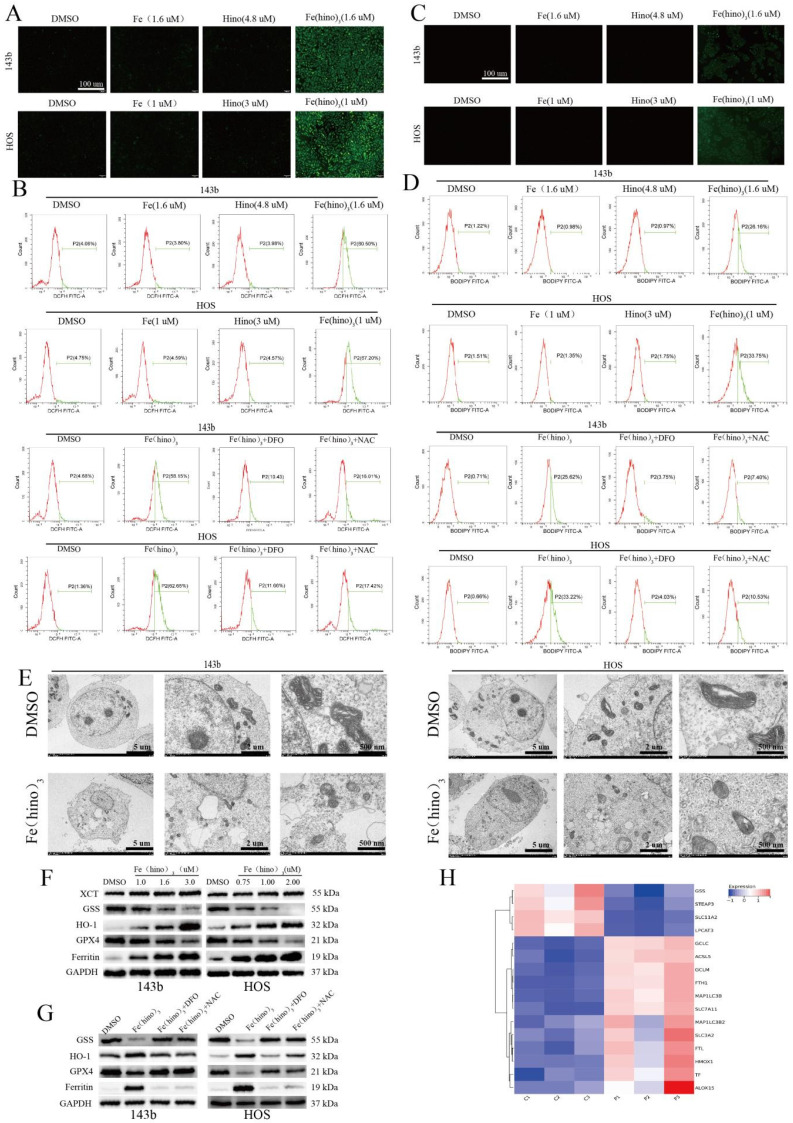
** Fe(hino)_3_ induced ferroptosis in osteosarcoma cells. (A)** Intracellular ROS levels detected by DCFH-DA probe. Fluorescence microscopy images showing ROS levels after 4 h treatment with FeCl3 (1.6 μM for 143b, 1 μM for HOS), hinokitiol (4.8 μM for 143b, 3 μM for HOS), or Fe(hino)_3_ (1.6 μM for 143b, 1 μM for HOS). Scale bar = 100 μm. **(B)** Flow cytometric quantification of intracellular ROS levels. The accumulation of ROS was reversed by pretreatment with DFO (50 μM, 6 h) or NAC (5 mM, 1 h). **(C)** Lipid peroxidation levels detected by Bodipy-C11 probe. Fluorescence microscopy images showing lipid peroxidation. Scale bar = 100 μm. **(D)** Flow cytometric quantification of lipid peroxidation. The accumulation was reversed by DFO and NAC pretreatment. **(E)** TEM images showing mitochondrial morphological changes after 24 h treatment with Fe(hino)3. Note the significant shrinkage of mitochondria. **(F-G)** Western blot analysis and quantification of ferroptosis-related proteins after 24 h treatment with Fe(hino)3 (1, 1.6, 3 μM for 143b; 0.75, 1, 2 μM for HOS). **(H)** RNA-seq showing changes in some ferroptosis-related genes after Fe(hino)3 (1.6 μM) treatment for 24 h in 143b cells.

JC-1 staining indicated a significant decrease in mitochondrial membrane potential (ΔΨm) after 12 hours of Fe(hino)_3_ treatment (Figure 3A), as quantified by flow cytometry, with this effect being reversed by DFO and NAC (Figure 3B). Western blot analysis confirmed a dose-dependent downregulation of mitochondrial respiratory chain complex I/II proteins, specifically SDHA and NDUFS2 (Figures 3C-D), which was also reversed by DFO and NAC (Figures 3E-F). The transcriptomic analysis identified a suppression of genes related to mitochondrial function following Fe(hino)_3_ exposure (Figures 3G). ROS levels, measured using the DCFH-DA probe, showed a significant increase 4 hours post-treatment (Figures 4A-B), and Bodipy-C11 staining revealed increased lipid peroxidation after 12 hours (Figures 4C-D). Transmission electron microscopy (TEM) demonstrated mitochondrial shrinkage in cells treated with Fe(hino)3 (Figure 4E). Western blotting further revealed that Fe(hino)_3_ treatment resulted in the suppression of GSS and GPX4, the upregulation of HO-1 and Ferritin, and a mild induction of XCT, all of which were reversed by DFO or NAC (Figures 4F-G). Transcriptomics also detected changes in some ferroptosis-related genes after treatment with Fe(hino)_3_ (Figure 4H).

### Fe(hino)_3_ induced pyroptosis via the NLRC4-caspase1-GSDMD axis

**Figure 5:**
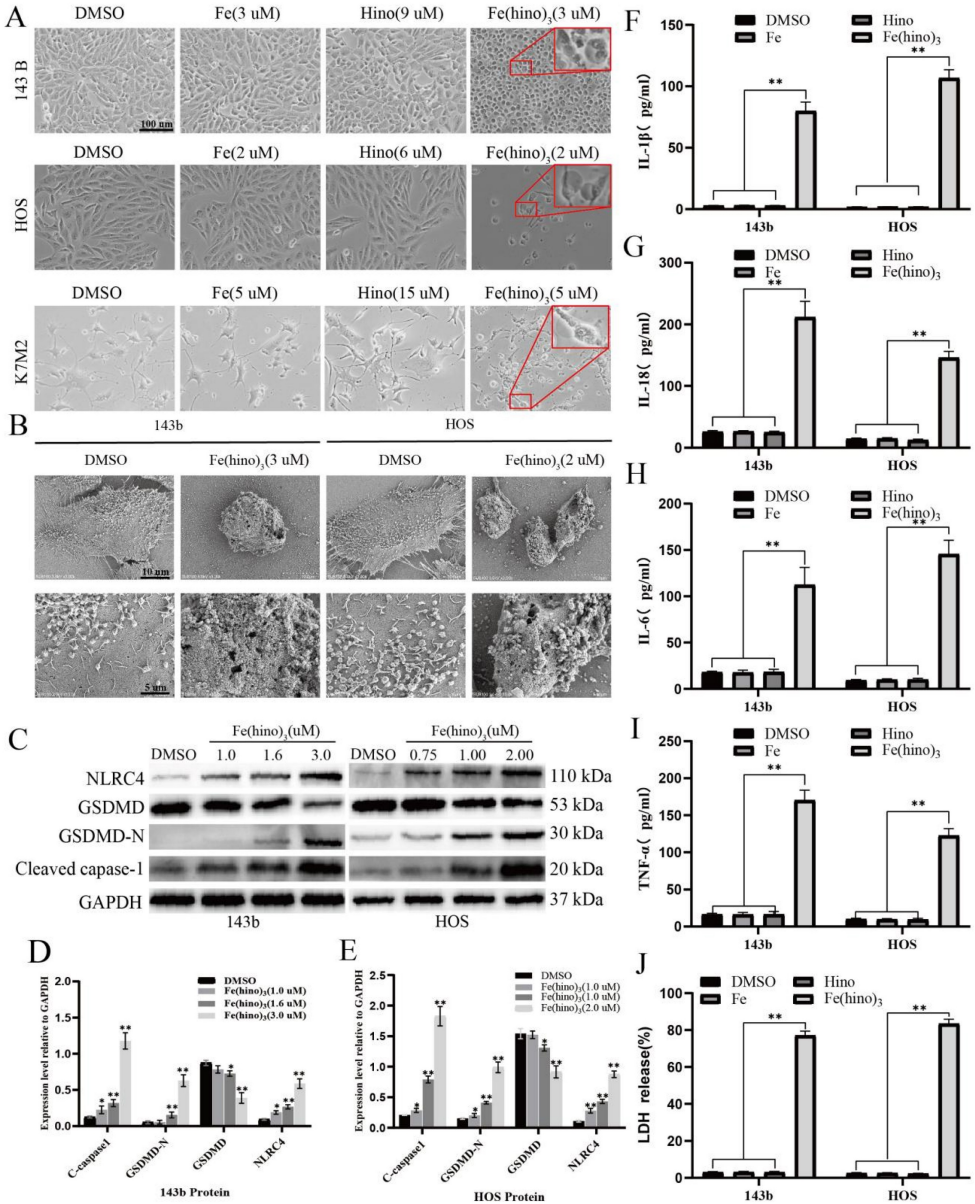
** Fe(hino)_3_ induced pyroptosis in osteosarcoma cells. (A)** Morphological changes observed under inverted microscope in 143b, HOS, and K7M2 cells after 24 h treatment with FeCl₃ (3 μM for 143b, 2 μM for HOS, 5 μM for K7M2), hinokitiol (9 μM for 143b, 6 μM for HOS, 15 μM for K7M2), or Fe(hino)₃ (3 μM for 143b, 2 μM for HOS, 5 μM for K7M2). **(B)** SEM images showing membrane swelling and pore formation after 24 h treatment with Fe(hino)3 (3 μM for 143b; 2 μM for HOS). **(C-E)** Western blot analysis of pyroptosis-related proteins (NLRC4, GSDMD-N, cleaved caspase-1) after 24 h treatment with Fe(hino)3 (1, 1.6, 3 μM for 143b; 0.75, 1, 2 μM for HOS). Quantification is shown in **(D, E)**. **(F-I)** ELISA quantification of inflammatory cytokines (IL-1β, IL-6, IL-18, TNF-α) in culture supernatants. **(J)** LDH release assay demonstrating membrane integrity loss. Data represent mean ± SD (n = 3). *P < 0.05 vs control, **0.01 < P < 0.05 vs control, ns represents no significance.

**Figure 6:**
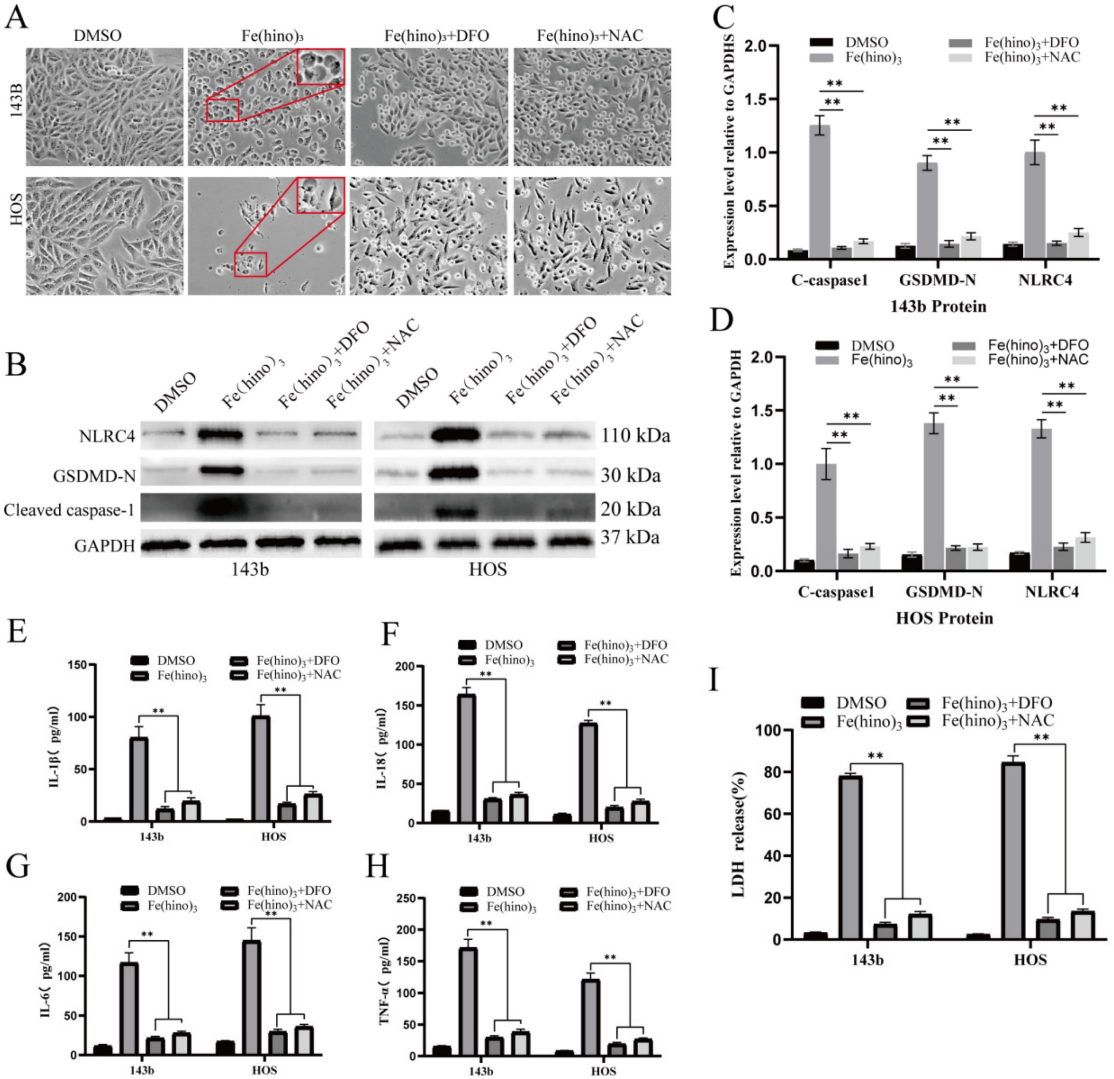
** DFO and NAC reversed Fe(hino)_3_-induced pyroptosis. (A)** Cell morphology observed under an inverted microscope. DFO (50 μM, 6 h pretreatment) or NAC (5 mM, 1 h pretreatment) reversed cell swelling and blebbing induced by Fe(hino)3 (3 μM for 143b, 2 μM for HOS) after 24 h treatment. Scale bar = 100 μm. **(B)** Western blot analysis and quantification show that DFO and NAC pretreatment inhibited Fe(hino)_3_-induced upregulation of pyroptosis-related proteins (p-NLRC4, GSDMD-N, and cleaved-caspase1). **(C-D)** Western blot analysis and quantification show that DFO and NAC pretreatment inhibited Fe(hino)_3_-induced upregulation of pyroptosis-related proteins (p-NLRC4, GSDMD-N, and cleaved-caspase1). **(E-H)** ELISA quantification of inflammatory cytokines (IL-1β, IL-6, IL-18, TNF-α) in culture supernatants. **(I)** LDH release assay showing that DFO and NAC pretreatment reversed Fe(hino)3-induced membrane damage. Data represent mean ± SD (n = 3). *P < 0.05 vs control, **0.01 < P < 0.05 vs control, ns represents no significance.

**Figure 7:**
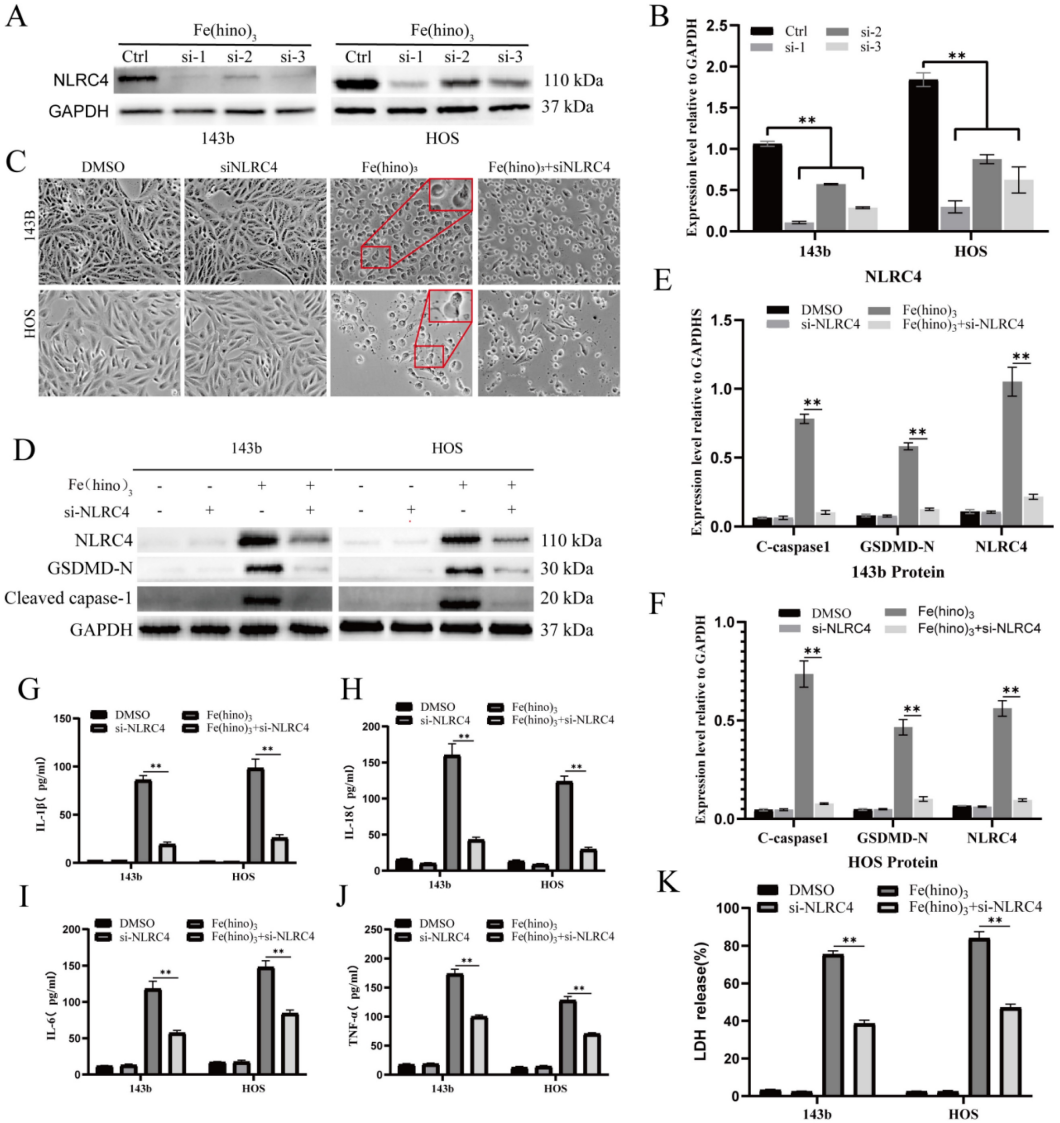
** NLRC4 knockdown attenuated Fe(hino)_3_-induced pyroptosis. (A-B)** Western blot analysis and quantification showing the knockdown efficiency of different si-NLRC4 sequences in osteosarcoma cells. **(C)** Cell morphology observed under an inverted microscope. si-NLRC4 (100 nM, 72 h pretreatment) attenuated morphological changes induced by Fe(hino)_3_ (3 μM for 143b, 2 μM for HOS) after 24 h treatment. Scale bar = 100 μm. **(D-F)** Western blot analysis and quantification showing that NLRC4 knockdown partially reversed Fe(hino)3-induced upregulation of GSDMD-N and cleaved-caspase1. **(G-J)** ELISA analysis showing that targeting NLRC4 partially reversed Fe(hino)_3_-induced elevation of inflammatory cytokines (IL-1β, IL-6, IL-18, TNF-α) in cell culture supernatants. **(K)** LDH release assay showing that targeting NLRC4 partially reversed Fe(hino)3-induced membrane damage. Data represent mean ± SD (n = 3). *P < 0.05 vs control, **0.01 < P < 0.05 vs control, ns represents no significance.

**Figure 8:**
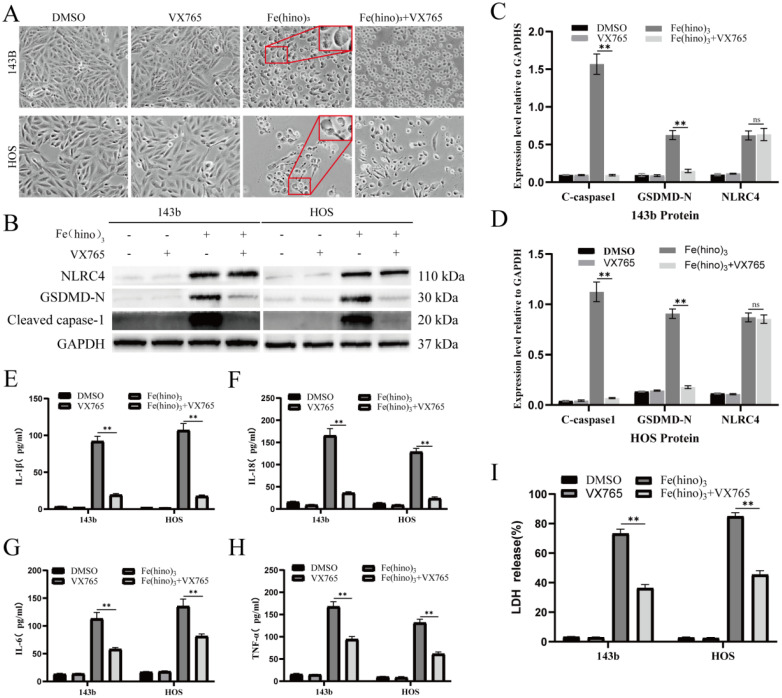
** Caspase-1 inhibition reduced Fe(hino)_3_-induced pyroptosis. (A)** Cell morphology observed under an inverted microscope. VX765 (30 μM, 1 h pretreatment) attenuated morphological changes induced by Fe(hino)_3_ (3 μM for 143b, 2 μM for HOS) after 24 h treatment. Scale bar = 100 μm. **(B)** Western blot analysis showing that Caspase1 inhibition partially reversed Fe(hino)3-induced upregulation of GSDMD-N and cleaved-caspase1, while having no significant effect on NLRC4 expression. **(C-D)** Western blot quantification of pyroptosis-related protein expression. **(E-H)** ELISA analysis showing that Caspase1 inhibition partially reversed Fe(hino)3-induced elevation of inflammatory cytokines (IL-1β, IL-6, IL-18, TNF-α) in cell culture supernatants. **(I)** LDH release assay showing that Caspase1 inhibition partially reversed Fe(hino)3-induced membrane damage. Daa represent mean ± SD (n = 3). *P < 0.05 vs control, **0.01< P < 0.05 vs control, ns represents no significance.

**Figure 9:**
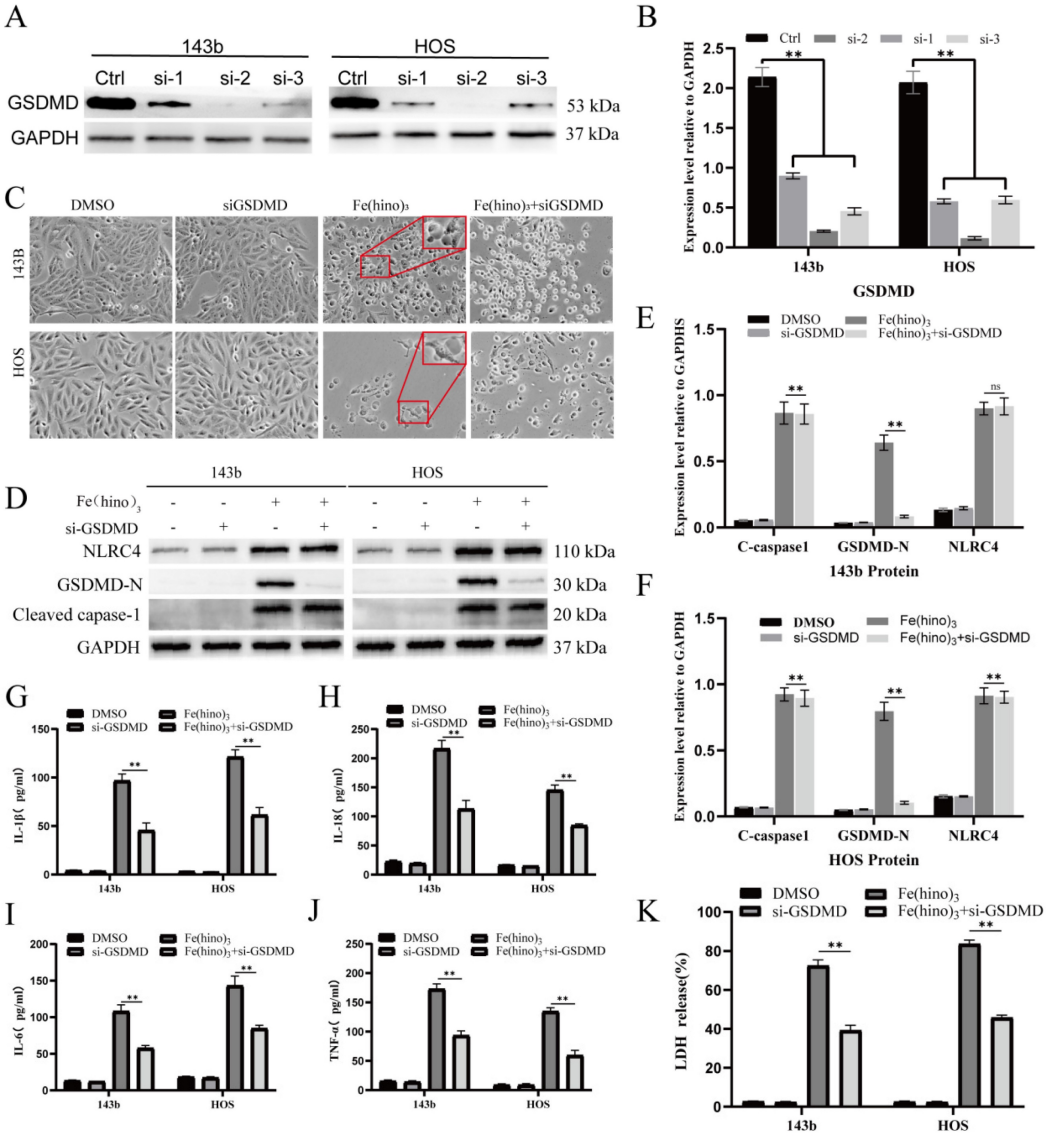
** GSDMD knockdown mitigated Fe(hino)_3_-induced pyroptosis. (A-B)** Western blot analysis and quantification showing the knockdown efficiency of different si-GSDMD sequences in osteosarcoma cells. **(C)** Cell morphology observed under an inverted microscope. si-GSDMD (100 nM, 72 h pretreatment) attenuated morphological changes induced by Fe(hino)3 (3 μM for 143b, 2 μM for HOS) after 24 h treatment. Scale bar = 100 μm. **(D-F)** Western blot and quantification of pyroptosis-related protein expression. **(G-J)** ELISA analysis showing that GSDMD knockdown partially reversed Fe(hino)3-induced elevation of inflammatory cytokines (IL-1β, IL-6, IL-18, TNF-α) in cell culture supernatants. **(K)** LDH release assay showing that GSDMD knockdown partially reversed Fe(hino)3-induced membrane damage. Data represent mean ± SD (n = 3). *P < 0.05 vs control.

The administration of high-dose Fe(hino)_3_ induced swelling in osteosarcoma cells (Figure 5A) and led to membrane blebbing accompanied by pore formation, as demonstrated through SEM (Figure 5B). Western blot analysis revealed an upregulation of pyroptosis-associated proteins, including phosphorylated NLRC4 (p-NLRC4), GSDMD-N, and cleaved caspase-1 (Figure 5C-E). Lactate dehydrogenase (LDH) release assays corroborated the occurrence of membrane rupture (Figure 5J), while enzyme-linked immunosorbent assays (ELISA) indicated increased levels of interleukin-1β (IL-1β), interleukin-18 (IL-18), interleukin-6 (IL-6), and tumor necrosis factor-alpha (TNF-α) (Figure 5F-I). Pretreatment with DFO or NAC mitigated the observed morphological alterations (Figure 6A), diminished the expression of pyroptosis-related proteins (Figure 6B-D), reduced LDH release (Figure 6I), and decreased cytokine secretion (Figure 6E-H). Similarly, knockdown of NLRC4 resulted in reduced cell swelling (Figure 7A-C), suppression of cleaved caspase-1 and GSDMD-N expression (Figure 7D-F), and a decrease in LDH (Figure 7K) and cytokine levels (Figure 7G-J). Inhibition of caspase-1 using VX765 and knockdown of GSDMD produced analogous effects (Figures 8-9).

### Fe(hino)3 suppressed tumor growth and enhanced immune infiltration *in vivo*

**Figure 10:**
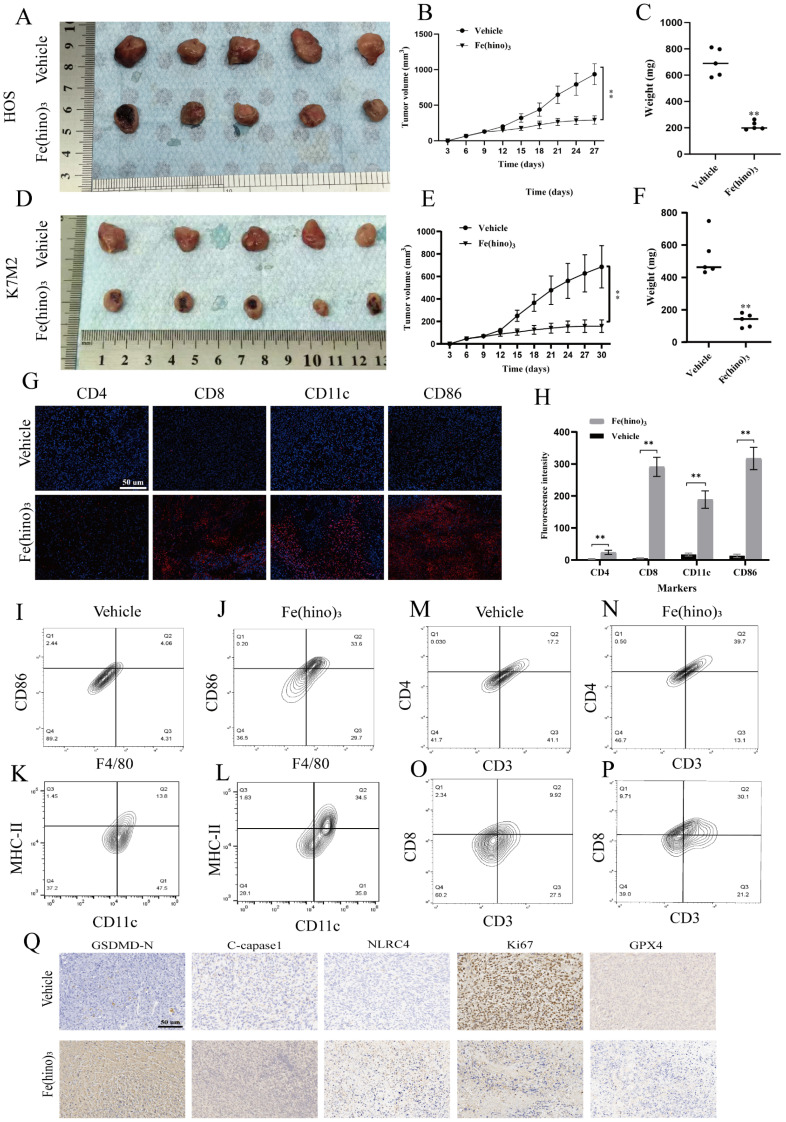
** Fe(hino)_3_ suppressed osteosarcoma growth *in vivo*. (A-C)** Representative images of HOS xenograft tumors. Mice (n=5 per group) received an intratumoral injection of Fe(hino)3 (50 μg) or vehicle when tumor volume reached approximately 70 mm³, with a second dose administered 5 days later: **(A)** Representative tumor specimens, **(B)** Tumor sizes, Volume = ½ × (short diameter)² × long diameter), and **(C)** Weights. **(D-F)** Representative images of K7M2 syngeneic model in Balb/c mice: (D) Tumor specimens, (E) Sizes, and (F) Weights. **(G)** Immunofluorescence staining showing infiltration of CD8+, CD11c+, CD86+, and CD4+ immune cells in K7M2 tumor tissues after Fe(hino)3 treatment. Scale bar = 50 μm. **(H)** Quantification of fluorescence intensity. **(I-P)** Flow cytometry analysis of M1 macrophage, DC cells, CD4^+^ T cells and CD8^+^ T cells in control and treated tumors. **(Q)** Immunohistochemical analysis of NLRC4, cleaved-caspase1, GSDMD-N, Ki67, and GPX4 expression in K7M2 tumor tissues.

In the HOS xenograft and K7M2 syngeneic models, the intratumoral administration of Fe(hino)_3_ resulted in significant inhibition of tumor growth, as depicted in Figure 10A-F. Immunofluorescence analysis demonstrated an increased infiltration of CD86⁺, CD11c⁺, CD8⁺, and CD4⁺ immune cells within the treated tumors (Figure 10G-H). Similarly, Flow cytometry suggested a significant increase in the proportion of M1 macrophages, DC cells, CD4^+^ T cells, and CD8^+^ T cells in the treated tumors (Figure 10I-P). Immunohistochemical assays confirmed the upregulation of NLRC4, cleaved-caspase1, and GSDMD-N, along with the downregulation of Ki67 and GPX4 (Figure 10Q). Increased expression of NLRC4, cleaved-caspase1, and GSDMD-N following the action of Fe(hino)3 revealed the activation of pyroptosis in tumors, which may serve as one of the important mechanisms of immune activation *in vivo*.

**Figure 11:**
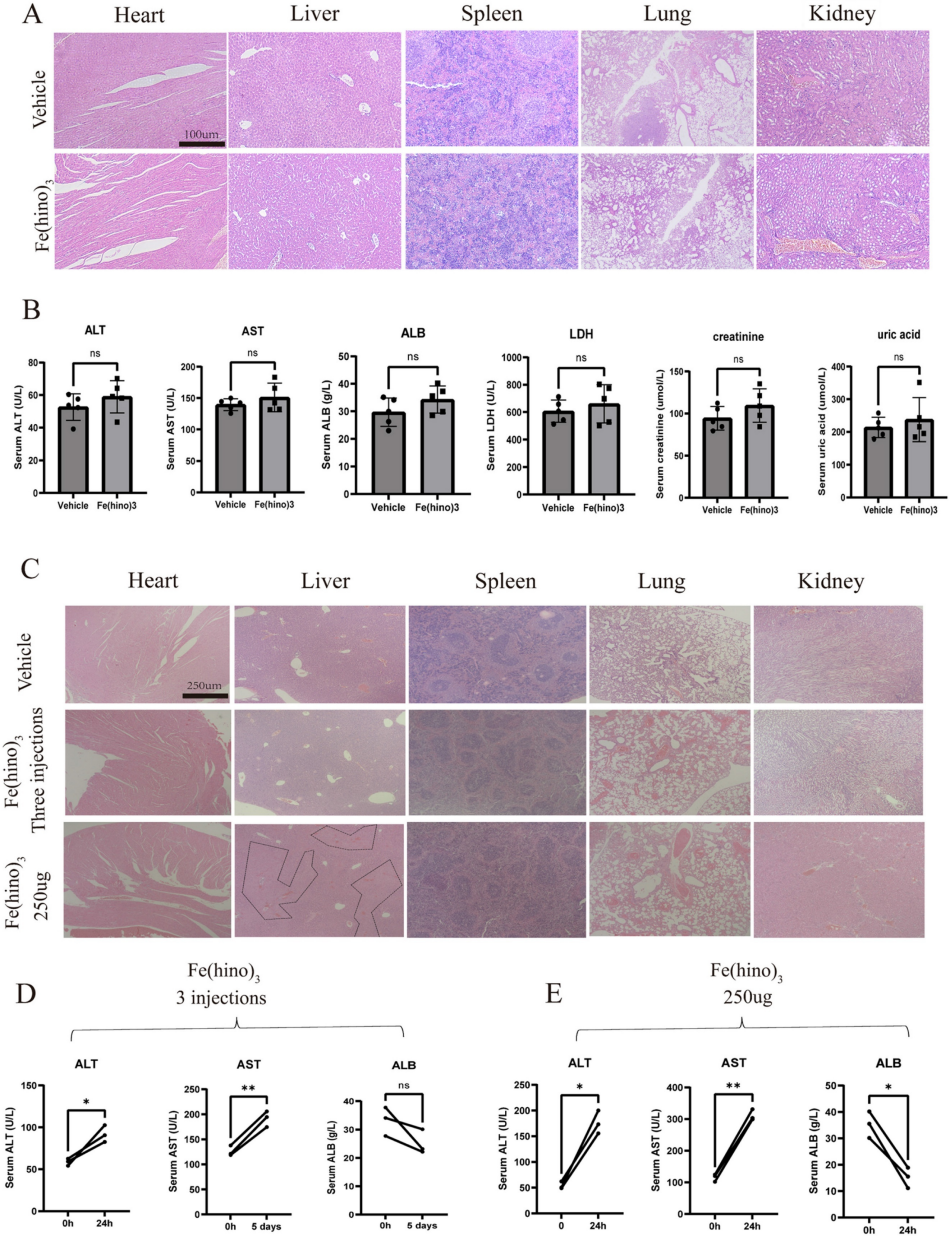
** Biosafety assessment of Fe(hino)_3_. (A)** H&E staining showing the effects of two local Fe(hino)_3_ injections on major organs (heart, liver, spleen, lung, kidney) in mice. Scale bar = 100 μm. **(B)** Assessment of serum biochemical indicators after two local Fe(hino)3 injections (50 ug). Ns represents no significant difference. **(C)** H&E staining of major organs after 3 injections of Fe(hino)_3_ and a single injection of 250 ug Fe(hino)_3_. **(D, E)**. Assessment of serum biochemical indicators at 5 days after 3 injections of Fe(hino)_3_ and 24h after a single injection of 250 ug Fe(hino)_3_. Ns represents no significant difference. *P < 0.05, **P < 0.01 vs control group.

Furthermore, H&E staining indicated the absence of major organ toxicity (Figure 11A) and serum biochemistry suggested no obvious markers of organ damage (Figure 11B). No significant side effects were observed in mice during the treatment period. To further assess the biosafety, we further performed 3 intratumoral injections (50ug per dose and each separated by 5 days). At the end of the treatment, H&E staining of the major organs suggested no obvious pathologic damage (Figure 11C). 5 days after the last injection, the serum levels of Alanine Transaminase (ALT) and Aspartate Aminotransferase (AST) were significantly increased compared with those before treatment, while Albumin (ALB) did not change significantly (Figure 11D). No other significant side effects were observed during the treatment. The above results suggested that 3 repeated applications of 50ug Fe(hino)3 might increase blood biochemical markers associated with liver injury, but not enough to cause pathologic damage in mice. To further investigate the maximum dose of Fe(hino)3 in a single injection, mice were further injected with 100ug, 150ug, 200ug and 250ug Fe(hino)3 intratumoral. The mice survived for more than 3 weeks at the 100ug, 150ug and 200ug doses but reduced mouse activity was observed in the 200ug dose group compared to the 50ug, 100ug and 150ug groups. The mice died at 72-96h after the application of 250g of Fe(hino)3. HE staining of the main organs showed that the liver was deformed and necrotic, while no significant toxicity was found in other organs (Figure 11C). The significant increase in serum ALT and AST and decrease in ALB at 24 h after injection suggested that the death of mice caused by the application of 250 g of Fe(hino)3 might be due to acute liver injury (Figure 11E).

### *In vivo* distribution of Fe(hino)3 injections

**Figure 12:**
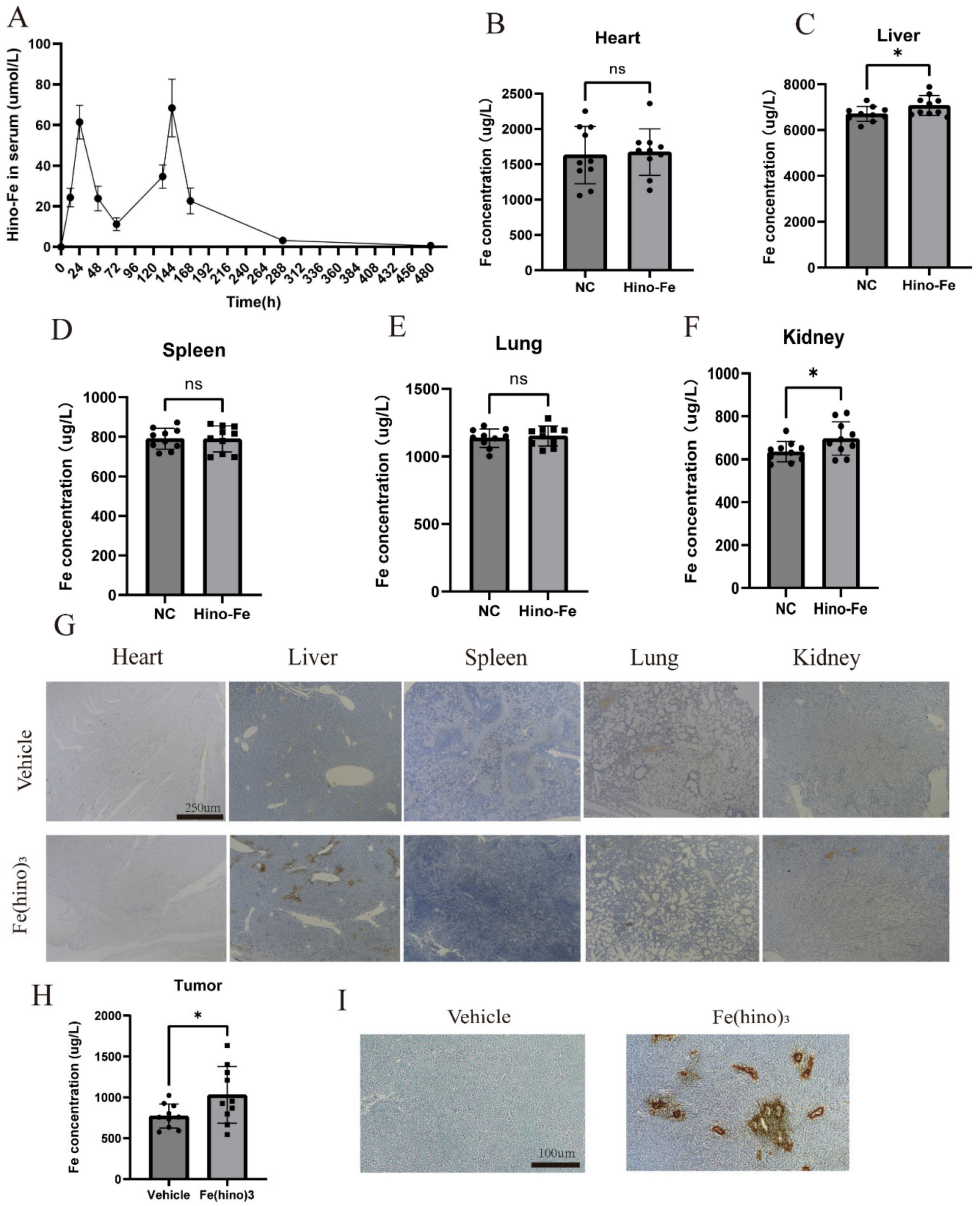
***In vivo* distribution of Fe(hino)_3_ by local injection. (A)** Blood concentration-time variation for 2 local injections of Fe(hino)_3_. **(B-F)** ICP-MS results of iron in major organs (heart, liver, spleen, lungs, kidneys) after 36 h of 50 ug Fe(hino)3 injection. Ns represents no significant difference. *P < 0.05 vs control, **0.01 < P < 0.05 vs control, ns represents no significance. **(G)** Prussian blue staining of major organs (heart, liver, spleen, lungs, kidneys) after 36 h of local 50 ug Fe(hino)_3_ injection. **(H)** ICP-MS of iron in the treated tumor after 36 h of 50ug Fe(hino)_3_ injection. **(I)** Prussian blue staining of tumors of different groups after 36 h of 50 ug Fe(hino)_3_ injection.

In the HOS xenograft model, the blood concentration-time folding plot of 2 intratumoral injections of Fe(hino)3 was shown in Figure 12A. Intratumoral injection reached peak blood concentration at about 24h, and 75% of the blood drug was metabolized at 72 h. To examine the visceral distribution of the local injection of Fe(hino)_3_, ICP-MS was used to measure the iron content in the visceral tissues at 36h after injection. Compared with the normal group, the iron content in the liver and kidney tissues of the treated group was significantly higher, while there was no significant change in other organs (Figure 12B-F). Prussian blue staining also suggested that iron deposition in the liver and kidney was significantly increased in the treatment group (Figure 12G). The iron content was also found be significantly increased in the treated tumors compared to the vehicle group (Figure 12H-I). The above results suggested that Fe(hino)3 was mainly distributed in the liver and kidney and caused a significant increase in iron content within the tumor after intratumoral injection.

## Discussion

In recent years, a multitude of studies have highlighted the therapeutic potential of metal complexes in cancer treatment 26. Hinokitiol, a natural monoterpenoid extracted from the heartwood of Cupressaceae family plants, exhibits broad-spectrum antitumor activity that surpasses its well-documented antimicrobial properties 27, 28. Although the anticancer efficacy of its iron complex, Fe(hino)₃, has been established in epithelial tumors, such as triple-negative breast cancer 5, its mechanism of action in mesenchymal-derived osteosarcoma remains inadequately understood. This study is the first to demonstrate that Fe(hino)₃ impedes the progression of osteosarcoma by inducing apoptosis, ferroptosis, and pyroptosis.

Our study demonstrated that Fe(hino)₃ markedly reduced the viability and clonogenicity of osteosarcoma cell lines (143b, HOS, and K7M2) at lower IC₅₀ concentrations compared to hinokitiol alone. Prussian blue staining revealed a significant accumulation of intracellular iron following treatment, which likely contributed to its enhanced antitumor efficacy. Intervention experiments with cell death inhibitors showed that at moderate doses (IC₅₀), the iron chelator DFO, ROS scavenger NAC, ferroptosis inhibitor Fer-1, and apoptosis inhibitor z-VAD-FMK significantly attenuated the cytotoxic effects induced by Fe(hino)₃. In contrast, at higher doses (IC₈₀), only DFO, NAC, Fer-1, and the caspase-1 inhibitor VX765 provided protective effects, indicating a dose-dependent activation of distinct cell death pathways.

Iron is integral to mitochondrial function, particularly in electron transport, and its dysregulation can result in mitochondrial dysfunction 29. Transcriptomic analysis indicated that Fe(hino)₃ significantly downregulated genes associated with mitochondrial function. JC-1 staining and flow cytometry revealed a marked decrease in mitochondrial membrane potential following Fe(hino)₃ treatment. Western blot analysis further corroborated a dose-dependent inhibition of respiratory chain complex I/II proteins, specifically NDUFS2^30^ and SDHA 31). Our previous studies had associated mitochondrial dysfunction with apoptosis 32 and ferroptosis 33 in osteosarcoma cells. Ferroptosis, an iron-dependent regulated cell death characterized by lipid peroxidation, has emerged as a promising strategy to overcome therapeutic resistance 34. The Fenton reaction, involving iron and hydrogen peroxide, produces reactive oxygen species 35, whereas the GSH/GPX4 antioxidant system mitigates oxidative damage 36. In the present study, treatment with Fe(hino)₃ resulted in the accumulation of ROS and lipid peroxidation, which was associated with mitochondrial shrinkage as observed through TEM. Western blot analysis revealed that Fe(hino)₃-induced downregulation of GSS and GPX4 was concomitant with the upregulation of HO-1 and Ferritin. These effects were reversed by the administration of DFO or NAC. Collectively, these findings suggested that Fe(hino)₃ induced ferroptosis in osteosarcoma cells via iron-ROS-mediated oxidative stress.

Zhang et al. have reported that oxidative stress could concurrently induce both ferroptosis and pyroptosis in osteosarcoma cells 37. In a similar vein, Tang et al. have demonstrated that mitochondrial reactive oxygen species (ROS) activated pyroptosis through the caspase-1-GSDMD pathway 38. Pyroptosis, which is characterized by cell swelling, membrane rupture 39, and the release of pro-inflammatory cytokines 40, was unexpectedly observed in osteosarcoma cells subjected to high-dose Fe(hino)₃ at IC80. SEM provided evidence of membrane blebbing and pore formation. Furthermore, Liu et al. have observed that the accumulation of iron ions within cells is associated with brain damage in a cerebral hemorrhage model 41. Simultaneously, research conducted by Chuanzhi Duan and Jin et al. had indicated that the NLRC4-caspase1-GSDMDN axis facilitates glial pyroptosis within the same experimental model 42, 43. Consequently, we hypothesize that the accumulation of iron ions may contribute to the mediation of this process. From a mechanistic perspective, exposure to high doses of Fe(hino)₃ resulted in the upregulation of NLRC4, GSDMD-N, and cleaved caspase-1, while ELISA assays revealed the increased release of LDH and pro-inflammatory cytokines, including IL-1β, IL-18, IL-6, and TNF-α. Interventions with DFO and NAC, as well as targeted inhibition of the NLRC4-caspase1-GSDMD axis, were observed to partially mitigate the cell death and inflammatory factor release triggered by elevated concentrations of Fe(hino)₃. These findings suggested that the iron-ROS-NLRC4-caspase1-GSDMD pathway was integral to the induction of pyroptosis in osteosarcoma cells subjected to increased levels of Fe(hino)_3_.

In addition to directly causing cell death, Fe(hino)₃ may affect anti-tumour immunity through several pathways. Fe(hino)₃ may activate dendritic cells (DCs) and cytotoxic T-lymphocytes (CTLs) by inducing ferroptosis, leading to phospholipid peroxidation and oxidative stress in tumor cell membranes and releasing damage-associated molecular patterns (DAMPs) 44. These mechanisms enhanced the response to immunotherapy. Decreased intra-T cell iron content has been found to mediate apoptosis, whereas Fe(hino)₃ may promote T cell infiltration in the tumor microenvironment by maintaining homeostasis of iron metabolism in T cells 11. Iron overload has been found to promote macrophage polarization towards a pro-inflammatory phenotype M1. Therefore, Fe(hino)₃ may also enhance anti-tumour immunity by directly increasing intracellular iron content in macrophages and promoting their polarization towards M1^12^. In addition to affecting anti-tumor immunity through the regulation of iron metabolism, hinokiol may also play a regulatory role. Hinokiol has been found to down-regulate the AKT/mTOR pathway, which can act as an upstream pathway for PD-L1 molecules 9 and thus shows potential for use in combination with immune checkpoint inhibitors. Amirmoezz Y et al. found that blocking mTOR in oral cancer up-regulated the expression of several immune accessory and adhesion molecules on the cell surface, including CD40, CD83, PD-L1, PD-L2, MHC class I, p selectin and VCAM-1^45^. In addition, mTOR acts as a center of metabolic regulation, activating downstream signaling pathways (e.g. PI3K-AKT) via the mTORC1 and mTORC2 complexes, driving metabolic reprogramming of tumor cells in terms of glucose uptake, essential amino acid utilization and lipid synthesis 46. This metabolic alteration leads to nutrient competition and metabolic waste accumulation (e.g., lactate, ROS) in the tumor microenvironment (TME), which suppresses T and NK cell function 47. Therefore, the application of Fe(hino)_3_ may also enhance the efficacy of immunotherapy due to the blocking effect of hinokiol on the AKT/mTOR pathway. In the present study, relatively high doses of Fe(hino)_3_ induced pyroptosis in osteosarcoma. The expression of inflammatory factors in downstream of pyroptosis was found to be significantly increased by Elisa assay. *In vivo*, the infiltration of CD4^+^ T cells, CD8^+^ T cells, DC cells and M1-type macrophages was increased after the action of Fe(hino)_3_. Therefore, in this study, pyroptosis-mediated immune activation of osteosarcoma may be one of the potential mechanisms. However, in osteosarcoma, the immune activation effect caused by the inhibition of AKT/mTOR by Fe(hino)₃ remains unclear, which is worthy of further exploration in the future.

In all, osteosarcoma is categorized as an immunologically "cold" tumor, which constrains the effectiveness of immunotherapy 48. In both HOS xenograft and K7M2 syngeneic models, Fe(hino)₃ showed a significant suppression of tumor growth. Immunofluorescence and immunohistochemistry analyses indicated an increased infiltration of CD86⁺, CD11c⁺, CD8⁺, and CD4⁺ immune cells, accompanied by the upregulation of NLRC4, cleaved caspase-1, and GSDMD-N, as well as the downregulation of Ki67 and GPX4. H&E staining and blood biochemistry revealed no significant organ toxicity, thereby affirming the safety profile of Fe(hino)₃. These results implied that Fe(hino)₃-induced immunogenic cell death might alter the immunosuppressive tumor microenvironment, thereby enhancing antitumor immunity. It's worth noting that due to the poor solubility and stability of Fe(hino)3 in water and fair solubility and stability in organic solvents, the intratumoral injections were used *in vivo*. This also suggests that the direct intratumoral injection of Fe(hino)3 may be more appropriate after future clinical translation. How to improve the water solubility of Fe(hino)3 for intravenous administration deserves further study in the future. Through pharmacological experiments, it was found that 72h after intratumoral injection, 75% of the blood drug was cleared. At the endpoint of treatment, H&E staining and blood biochemistry did not reveal significant organ toxicity or side effects. Three repeated injections altered only the blood biochemical parameters of the liver without causing pathological damage. The maximum tolerated dose of Fe(hino)3 in mice is approximately 5 times of the therapeutic dose. These results are suggestive of good biosafety and good clinical transfer potential of Fe(hino)3. More importantly, Fe(hino)3 mediated cell death in a dose-dependent manner. Relatively high doses produced more pronounced pyroptosis compared to relatively low doses. This suggests that when considering Fe(hino)3 as an alternative to conventional chemotherapy or targeted therapy agents, relatively low doses may be adequate to improve biosafety and reduce potential side effects. However, when considering its use in combination with immunotherapy, a relatively safe and high dose should be employed to enhance the efficacy of immunotherapy due to pyroptosis-mediated immune activation.

Notwithstanding the encouraging results, this study is subject to several limitations: (1) the mechanisms responsible for the selectivity of Fe(hino)₃ towards osteosarcoma cells have yet to be elucidated; (2) the relative contributions of ferroptosis and pyroptosis to its antitumor effects necessitate further quantification; (3) the potential synergistic effects of Fe(hino)₃ in combination with existing therapies warrant investigation. Future research should address these issues to advance the clinical translation of Fe(hino)₃.

## Conclusion

This research is the inaugural study to illustrate that Fe(hino)₃ inhibited osteosarcoma proliferation via the dual induction of ferroptosis and pyroptosis. This distinctive mechanism not only directly eradicated tumor cells but also augmented antitumor immunity by restructuring the tumor microenvironment. Notably, the present study found that Fe(hino)₃ mediated osteosarcoma cell death in a dose-dependent manner. At relatively low doses, Fe(hino)₃ induced ferroptosis primarily in tumor cells. The possible hepatotoxicity of Fe(hino)₃ was shown in subsequent animal experiments. At relatively high doses, Fe(hino)₃ induced pyroptosis in tumor cells, which in turn induced anti-tumor immune activation. Therefore, in clinical applications, relatively high doses of Fe(hino)₃ in combination with immunotherapy may result in greater benefits due to stronger Fe(hino)₃-mediated immune activation, but potential hepatotoxicity still needs to be considered. In the future, the appropriate dosage of Fe(hino)₃ for combination with commonly used immunotherapies such as anti-PD-L1 treatment deserves further exploration, while the biological safety should also be evaluated simultaneously although adequate immune activation had been observed in animals at relatively safe doses in this study. These results offer a theoretical basis for the development of innovative therapeutic strategies targeting refractory osteosarcoma.

## Figures and Tables

**Figure 13:**
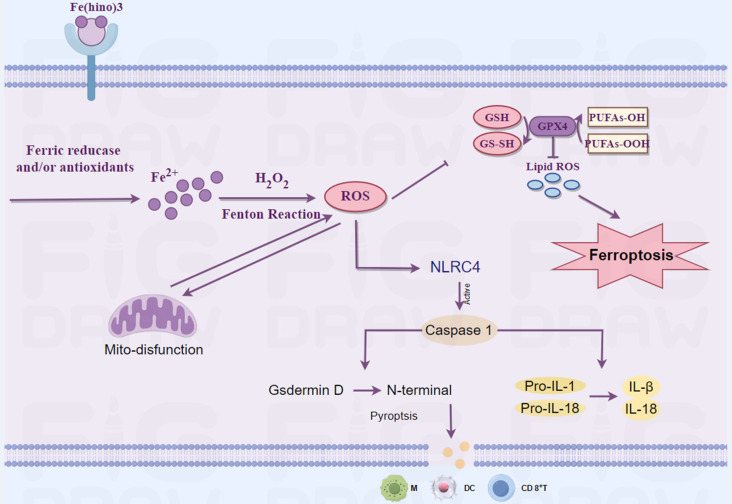
** A schematic representation of the mechanism through which Fe(hino)3 inhibits osteosarcoma.** This figure was drawn by Figdraw.

## References

[1] Tian H, Cao J, Li B, Nice EC, Mao H, Zhang Y (2023). Managing the immune microenvironment of osteosarcoma: the outlook for osteosarcoma treatment. Bone Research.

[2] Shi P, Cheng Z, Zhao K, Chen Y, Zhang A, Gan W (2023). Active targeting schemes for nano-drug delivery systems in osteosarcoma therapeutics. Journal of Nanobiotechnology.

[3] Chelpuri Y, Pabbathi S, Alla GR, Yadala RK, Kamishetti M, Banothu AK (2022). Tropolone derivative hinokitiol ameliorates cerulein-induced acute pancreatitis in mice. International Immunopharmacology.

[4] Liu S, Yamauchi H (2009). p27-Associated G1 arrest induced by hinokitiol in human malignant melanoma cells is mediated via down-regulation of pRb, Skp2 ubiquitin ligase, and impairment of Cdk2 function. Cancer Letters.

[5] Zhao H, Zhang M, Zhang J, Sun Z, Zhang W, Dong W (2023). Hinokitiol-iron complex is a ferroptosis inducer to inhibit triple-negative breast tumor growth. Cell & Bioscience.

[6] Li B, Zhang B, Cheng Z, Lou Y, Chen S (2025). Nanomaterials targeting iron homeostasis: a promising strategy for cancer treatment. Frontiers in Bioengineering and Biotechnology.

[7] Abeydeera N, Stilgenbauer M, Pant BD, Mudarmah K, Dassanayake TM, Zheng Y-R (2023). Lipophilic Fe(III)-Complex with Potent Broad-Spectrum Anticancer Activity and Ability to Overcome Pt Resistance in A2780cis Cancer Cells. Molecules.

[8] Cheriyan BV, Srinivasan P, Push GJ, K K K, Sainath PB, Shanmugam A (2024). *In silico* and *In vitro* Evaluation of Cytotoxic Potential of Hinokitiol against Osteosarcoma by targeting Glycogen Synthase Kinase 3β. Turkish Journal of Pharmaceutical Sciences.

[9] Wu P-S (2025). Hinokitiol reduces tumor metastasis by regulating epithelial cell adhesion molecule via protein kinase-B/mammalian target of rapamycin signaling pathway. American Journal of Cancer Research.

[10] Tang H, He K, Zhao K, Zheng C, Wu W, Jin W (2024). Protective Effects of Hinokitiol on Neuronal Ferroptosis by Activating the Keap1/Nrf2/HO-1 Pathway in Traumatic Brain Injury. Journal of Neurotrauma.

[11] Che R, Wang Q, Li M, Shen J, Ji J (2024). Quantitative Proteomics of Tissue-Infiltrating T Cells From CRC Patients Identified Lipocalin-2 Induces T-Cell Apoptosis and Promotes Tumor Cell Proliferation by Iron Efflux. Molecular & Cellular Proteomics.

[12] Bu X, Wang L (2024). Iron metabolism and the tumor microenvironment: A new perspective on cancer intervention and therapy (Review). International Journal of Molecular Medicine.

[13] Zhao P, Yin S, Qiu Y, Sun C, Yu H (2025). Ferroptosis and pyroptosis are connected through autophagy: a new perspective of overcoming drug resistance. Molecular Cancer.

[14] Tang R, Xu J, Zhang B, Liu J, Liang C, Hua J (2020). Ferroptosis, necroptosis, and pyroptosis in anticancer immunity. Journal of Hematology & Oncology.

[15] Jiang M, Jike Y, Liu K, Gan F, Zhang K, Xie M (2023). Exosome-mediated miR-144-3p promotes ferroptosis to inhibit osteosarcoma proliferation, migration, and invasion through regulating ZEB1. Molecular Cancer.

[16] Kang N, Son S, Min S, Hong H, Kim C, An J (2023). Stimuli-responsive ferroptosis for cancer therapy. Chemical Society Reviews.

[17] Dixon SJ, Olzmann JA (2024). The cell biology of ferroptosis. Nature Reviews Molecular Cell Biology.

[18] Tan Y, Chen Q, Li X, Zeng Z, Xiong W, Li G (2021). Pyroptosis: a new paradigm of cell death for fighting against cancer. Journal of Experimental & Clinical Cancer Research.

[19] Wang H, Wang T, Yan S, Tang J, Zhang Y, Wang L (2024). Crosstalk of pyroptosis and cytokine in the tumor microenvironment: from mechanisms to clinical implication. Molecular Cancer.

[20] Tong G, Shen Y, Li H, Qian H, Tan Z (2024). NLRC4, inflammation and colorectal cancer (Review). International Journal of Oncology.

[21] Pandey A, Li Z, Gautam M, Ghosh A, Man SM (2024). Molecular mechanisms of emerging inflammasome complexes and their activation and signaling in inflammation and pyroptosis. Immunological Reviews.

[22] Puth S, Verma V, Hong SH, Tan W, Lee SE, Rhee JH (2022). An all-in-one adjuvanted therapeutic cancer vaccine targeting dendritic cell cytosol induces long-lived tumor suppression through NLRC4 inflammasome activation. Biomaterials.

[23] Yu X, Liu W, Chen S, Cheng X, Paez PA, Sun T (2021). Immunologically programming the tumor microenvironment induces the pattern recognition receptor NLRC4-dependent antitumor immunity. Journal for ImmunoTherapy of Cancer.

[24] Feng Q, Qi F, Fang W, Hu P, Shi J (2024). Ferroptosis to Pyroptosis Regulation by Iron-Based Nanocatalysts for Enhanced Tumor Immunotherapy. Journal of the American Chemical Society.

[25] Wang Z, Tang Y, Li Q (2025). A self-assembling nanoplatform for pyroptosis and ferroptosis enhanced cancer photoimmunotherapy. Light: Science & Applications.

[26] Santa Maria de la Parra L, Balsa LM, León IE (2024). Metallocompounds as anticancer agents against osteosarcoma. Drug Discovery Today.

[27] Wu Y-J, Hsu W-J, Wu L-H, Liou H-P, Pangilinan CR, Tyan Y-C (2020). Hinokitiol reduces tumor metastasis by inhibiting heparanase via extracellular signal-regulated kinase and protein kinase B pathway. International Journal of Medical Sciences.

[28] Chiang Y-F, Huang K-C, Chen H-Y, Hamdy NM, Huang T-C, Chang H-Y (2024). Hinokitiol Inhibits Breast Cancer Cells *In vitro* Stemness-Progression and Self-Renewal with Apoptosis and Autophagy Modulation via the CD44/Nanog/SOX2/Oct4 Pathway. International Journal of Molecular Sciences.

[29] van der Meer P, van der Wal HH, Melenovsky V (2019). Mitochondrial Function, Skeletal Muscle Metabolism, and Iron Deficiency in Heart Failure. Circulation.

[30] Bandara AB, Drake JC, James CC, Smyth JW, Brown DA (2021). Complex I protein NDUFS2 is vital for growth, ROS generation, membrane integrity, apoptosis, and mitochondrial energetics. Mitochondrion.

[31] Dalla Pozza E, Dando I, Pacchiana R, Liboi E, Scupoli MT, Donadelli M (2020). Regulation of succinate dehydrogenase and role of succinate in cancer. Seminars in Cell & Developmental Biology.

[32] Huang Q, Ou Y-S, Tao Y, Yin H, Tu P-H (2016). Apoptosis and autophagy induced by pyropheophorbide-α methyl ester-mediated photodynamic therapy in human osteosarcoma MG-63 cells. Apoptosis.

[33] Zhang Y, Chen Y, Mou H, Huang Q, Jian C, Tao Y (2024). Synergistic induction of ferroptosis by targeting HERC1-NCOA4 axis to enhance the photodynamic sensitivity of osteosarcoma. Redox Biology.

[34] Zhang C, Liu X, Jin S, Chen Y, Guo R (2022). Ferroptosis in cancer therapy: a novel approach to reversing drug resistance. Molecular Cancer.

[35] Min H, Qi Y, Zhang Y, Han X, Cheng K, Liu Y (2020). A Graphdiyne Oxide-Based Iron Sponge with Photothermally Enhanced Tumor-Specific Fenton Chemistry. Advanced Materials.

[36] Wang X, Shen T, Lian J, Deng K, Qu C, Li E (2023). Resveratrol reduces ROS-induced ferroptosis by activating SIRT3 and compensating the GSH/GPX4 pathway. Molecular Medicine.

[37] Zhang L, Song A, Yang Q-C, Li S-J, Wang S, Wan S-C (2023). Integration of AIEgens into covalent organic frameworks for pyroptosis and ferroptosis primed cancer immunotherapy. Nature Communications.

[38] Jin J, Yuan P, Yu W, Lin J, Xu A, Xu X (2022). Mitochondria-Targeting Polymer Micelle of Dichloroacetate Induced Pyroptosis to Enhance Osteosarcoma Immunotherapy. ACS Nano.

[39] Elias EE, Lyons B, Muruve DA (2023). Gasdermins and pyroptosis in the kidney. Nature Reviews Nephrology.

[40] Yang F, Bettadapura SN, Smeltzer MS, Zhu H, Wang S (2022). Pyroptosis and pyroptosis-inducing cancer drugs. Acta Pharmacologica Sinica.

[41] Liu C, He P, Guo Y, Tian Q, Wang J, Wang G (2022). Taurine attenuates neuronal ferroptosis by regulating GABAB/AKT/GSK3β/β-catenin pathway after subarachnoid hemorrhage. Free Radical Biology and Medicine.

[42] Ding M, Jin L, Wei B, Cheng W, Liu W, Li X (2024). Tumor necrosis factor-stimulated gene-6 ameliorates early brain injury after subarachnoid hemorrhage by suppressing NLRC4 inflammasome-mediated astrocyte pyroptosis. Neural Regeneration Research.

[43] Jin P, Qi D, Cui Y, Lenahan C, Zhang JH, Tao X (2022). Aprepitant attenuates NLRC4-dependent neuronal pyroptosis via NK1R/PKCδ pathway in a mouse model of intracerebral hemorrhage. Journal of Neuroinflammation.

[44] Gu X, Liu Ye, Dai X, Yang Y-G, Zhang X (2023). Deciphering the potential roles of ferroptosis in regulating tumor immunity and tumor immunotherapy. Frontiers in Immunology.

[45] Yonesi A, Tomihara K, Takatsuka D, Tachinami H, Yamazaki M, Jadidi ARY (2024). Rapamycin Induces Phenotypic Alterations in Oral Cancer Cells That May Facilitate Antitumor T Cell Responses. Biomedicines.

[46] Glorieux C, Enríquez C, Buc Calderon P (2025). The complex interplay between redox dysregulation and mTOR signaling pathway in cancer: A rationale for cancer treatment. Biochemical Pharmacology.

[47] Ricci J-E (2025). Tumor-induced metabolic immunosuppression: Mechanisms and therapeutic targets. Cell Reports.

[48] Zhu T, Han J, Yang L, Cai Z, Sun W, Hua Y (2022). Immune Microenvironment in Osteosarcoma: Components, Therapeutic Strategies and Clinical Applications. Frontiers in Immunology.

